# Performance of chatbots in queries concerning fundamental concepts in photochemistry

**DOI:** 10.1111/php.14037

**Published:** 2024-11-04

**Authors:** Masahiko Taniguchi, Jonathan S. Lindsey

**Affiliations:** ^1^ Department of Chemistry North Carolina State University Raleigh North Carolina USA

**Keywords:** artificial intelligence, generative AI, machine learning, natural language processing, PhotochemCAD, search engines, spectra

## Abstract

The advent of chatbots raises the possibility of a paradigm shift across society including the most technical of fields with regard to access to information, generation of knowledge, and dissemination of education and training. Photochemistry is a scientific endeavor with roots in chemistry and physics and branches that encompass diverse disciplines ranging from astronomy to zoology. Here, five chatbots have each been challenged with 13 photochemically relevant queries. The chatbots included ChatGPT 3.5, ChatGPT 4.0, Copilot, Gemini Advanced, and Meta AI. The queries encompassed fundamental concepts (e.g., “Why is the fluorescence spectrum typically the mirror image of the absorption spectrum?”), practical matters (e.g., “What is the inner filter effect and how to avoid it?”), philosophical matters (“Please create the most important photochemistry questions.”), and specific molecular features (e.g., “Why are azo dyes non‐fluorescent?”). The chatbots were moderately effective in answering queries concerning fundamental concepts in photochemistry but were glaringly deficient in specialized queries for dyes and fluorophores. In some instances, a correct response was embedded in verbose scientific nonsense whereas in others the entire response, while grammatically correct, was utterly meaningless. The unreliable accuracy makes present chatbots poorly suited for unaided educational purposes and highlights the importance of domain experts.

## Graphical abstract

The advent of chatbots raises the possibility of a paradigm shift in many disciplines of scientific research. Here, 13 photochemically relevant queries were posed to five chatbots. The queries include fundamental concepts, practical or philosophical matters, and properties of dyes. The chatbot responses ranged from moderately effective to glaringly deficient, with instances of a correct response embedded in scientific nonsense or even entirely meaningless responses. Chatbots are complementary to search engines and may comprise a useful tool for scientific research in the hands of a domain expert. The unreliable accuracy makes present chatbots unsuited for unguided educational purposes in photochemistry.

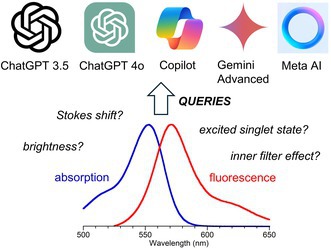

**Abbreviations**
AI: Artificial Intelligence
CAD: Computer‐Aided Design
FLIM: Fluorescence Lifetime Imaging Microscopy
FRET: Förster Resonance Energy Transfer
GPT: Generative Pre‐trained Transformer
LaMDA: Language Model for Dialogue Applications
LCD: Liquid Crystal Display
Llama: Large language model meta AI
LLMs: Large Language Models
Q#: Query number
QLED: Quantum Light‐Emitting Diode

## INTRODUCTION

Photochemistry is a foundational science that underpins numerous disciplines yet itself is often esoteric. The concepts of a quantum yield, excitation spectrum, and phosphorescence versus fluorescence, are not typically familiar to a graduate with a 4‐year chemistry degree. Graduate science education is quite specialized and many Ph.D. degree holders in chemistry, for example, also are unfamiliar with such topics. Individuals who encounter photochemistry through research or other activities nowadays likely turn to web‐based searches to identify salient points of information or to gain conceptual understanding.

Our experience with knowledge on the part of the non‐expert has come from a variety of activities. One of us (J.S.L.) has taught undergraduate chemistry and photochemistry for >4 decades and has delivered lectures to broad scientific audiences concerning artificial photosynthesis; in both activities, questions from students and scientists outside the field have proved enlightening. Both of us have been developing PhotochemCAD over several decades, during which numerous inquiries from researchers (graduate, professional, etc.) also have revealed common misunderstandings or even surprising lack of awareness of the aforementioned concepts. PhotochemCAD comprises curated databases of absorption and fluorescence spectra^1^, ^2^, ^3^, ^4^, ^5^, ^6^, ^7^, ^8^, ^9^ along with calculational modules^10^ for use in the fields of photochemistry and photobiology. The challenge of finding spectra and companion parameters (molar absorption coefficient, fluorescence quantum yield) in the published literature^11^ has led, inter alia, to the exploration of the use of artificial intelligence methods for mining the internet.

A relatively recent manifestation of artificial intelligence has arrived in the form of chatbots. Chatbots (namely, ChatGPT) are conversational‐styled, artificial intelligence web‐based graphical user interfaces that make use of the architecture of large language models (LLMs).^12^, ^13^, ^14^, ^15^, ^16^, ^17^, ^18^, ^19^, ^20^, ^21^, ^22^ Since 2023, the capability of ChatGPT to respond to domain‐specific queries has been extensively studied in diverse fields of science. Representative fields include general chemistry,^23^, ^24^, ^25^, ^26^, ^27^ clinical diagnostics,^28^, ^29^, ^30^, ^31^ engineering,^32^, ^33^ environmental sciences,^34^, ^35^, ^36^ inorganic chemistry,^37^, ^38^, ^39^ materials chemistry,^40^, ^41^ medicine,^42^, ^43^, ^44^, ^45^, ^46^ microbiology,^47^, ^48^ organic chemistry,^49^, ^50^ and pathology.^51^, ^52^ ChatGPT (and GPT models) have also been examined for medical and pharmacy licensing examinations^53^ to verify their performance, whereupon the accuracies to the queries were as follows for particular exams: United States medical (60% correct, passing threshold),^54^, ^55^ United Kingdom medical (76% correct, pass),^56^ Japanese medical (>87% correct, pass),^57^ Brazilian medical revalidation (88% correct, pass),^58^ Japanese pharmaceutical (>60% correct),^59^ and Taiwanese pharmaceutical (~55% correct, fail).^60^ The knowledge of chatbots relies on the training datasets applied for LLMs; thus, the availability and quality of the datasets are critical for generation of accurate responses. The datasets used for training of LLMs consist of C4 (Common Crawl's web crawl corpus), Books1 and Books2, dialog data from public forums, OpenWebText2, GitHub, Wikipedia, ArXiv, and PubMed abstracts.

**FIGURE 1:**
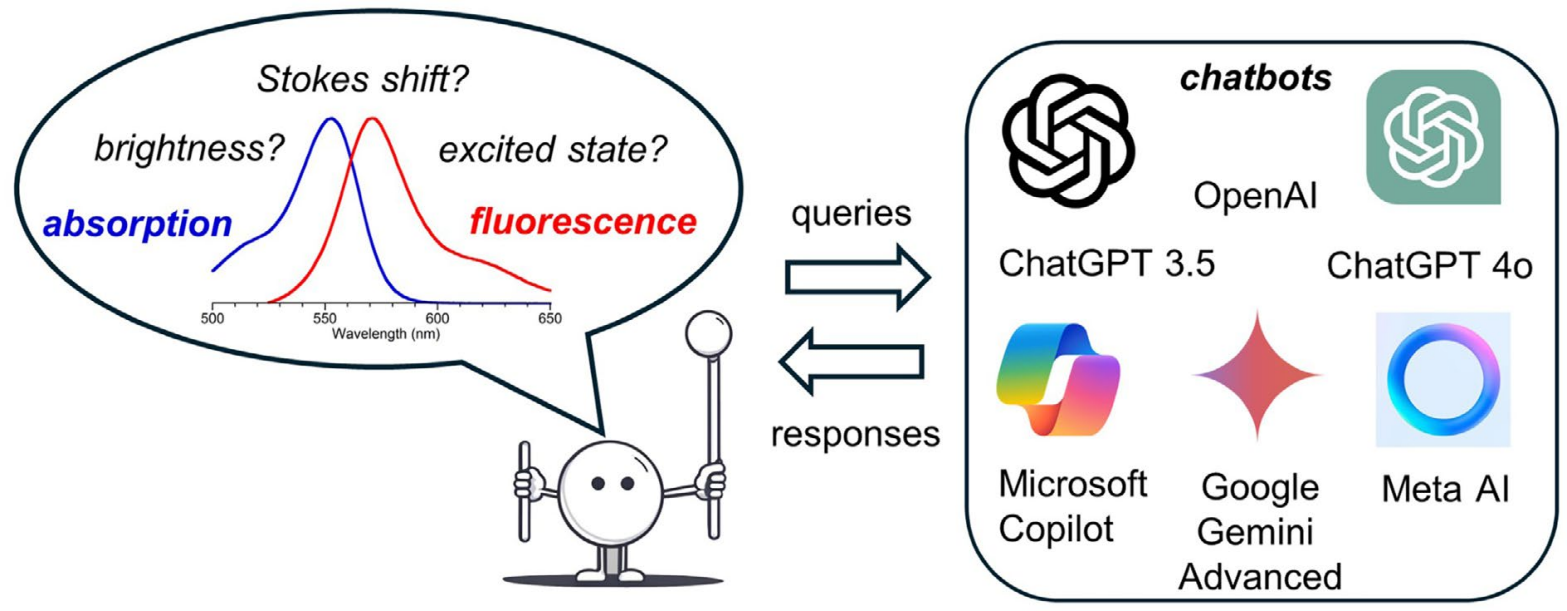
Queries to and responses from Chatbots concerning photochemistry topics.

The advent of chatbots raises the question whether the concepts that are well known to domain experts in the fields of photochemistry and photobiology can be accurately reported (Figure 1). In this paper, five chatbots have been examined to address queries concerning fundamental concepts in photochemistry. The queries encompassed scientific concepts that are typically addressed in the first few chapters of an introductory book on photochemistry. Additional queries addressed somewhat more specialized issues, although again the questions and answers were expected to be familiar to the wide variety of scientists with familiarity in photochemistry and photobiology.

## MATERIALS AND METHODS

The queries were addressed to five major chatbots and their variants (ChatGPT 3.5 and 4o; Copilot and Gemini Advanced; Meta AI) followed by manual analysis of the responses. ChatGPT 3.5, Copilot, and Meta AI are freely accessible through web interfaces, whereas ChatGPT 4o and Gemini Advance are subscription‐based paid platforms (~$20 per month). Copilot provides control over conversation styles with three different levels depending on the exactitude of the answer (creative, balanced, and precise); precise was chosen for the present study. Each chatbot has an underlying LLM: (i) ChatGPT 3.5 is based on GPT‐3.5 (Generative Pre‐trained Transformer);^15^ (ii) ChatGPT 4o is based on GPT‐4;^22^ (iii) Copilot is also based on GPT‐4 but incorporates further results from web searches; (iv) Gemini is the successor to Google's chatbot Bard,^61^, ^62^ which was based on LaMDA;^18^ and (v) Meta AI utilizes Llama.^21^

Chat GPT 3.5 and 4o rarely display web links corresponding to their response contexts, thwarting deeper analysis by the human domain expert. Copilot and Meta AI often provide web links, but citations and footnote links are not always appropriately suited. Furthermore, the generation of links that are poorly correlated (i.e., pointing to digressive, unrelated material) is observed in some cases. Gemini Advance has a “double‐check response” button to search on the web to find context similar to their own response and provide web links (when the context is available on the web).

To examine the ability of chatbots to respond concerning knowledge in photochemistry, thirteen queries were posed. All the queries were made from a single user account in each platform only once per query in the period May 24–26 of 2024, in an effort to keep the conditions uniform for the responses. The queries are listed in Table 1.

**TABLE 1:** Photochemically relevant queries.

**Fundamental concepts**	
Q1	Why is the fluorescence spectrum typically the mirror image of the absorption spectrum?
Q2	What is the difference between the absorption spectrum and the excitation spectrum?
Q3	What is the role of the lowest vibrational level of the excited singlet state in photochemistry?
Q4	Why is the Stokes shift relevant?
Q5	Why is the excited triplet state generally longer‐lived than the excited singlet state?
Q6	Why is (or is not) the lifetime of an excited state important?
Q7	Why does the fluorescence quantum yield of a fluorophore decrease as the fluorescence spectrum shifts to a longer wavelength?
Q8	Why is the quantum yield of phosphorescence generally low for organic compounds in solution?
**Practical matter**	
Q9	What is the inner filter effect and how to avoid it?
**Philosophical issue**	
Q10	Please create the most important photochemistry questions
**Dyes and fluorophores**	
Q11	Why are azo dyes non‐fluorescent?
Q12	Why do porphyrins typically exhibit two peaks in the fluorescence spectrum?
Q13	Why do quantum dots typically exhibit high brightness?

## RESULTS AND DISCUSSION

The full response by each chatbot to each query is provided in the Appendix S1. In the following, the queries and our evaluation of the responses are provided. The evaluations are drawn from knowledge of basic photochemistry as provided in classic texts.^63^, ^64^, ^65^, ^66^, ^67^, ^68^, ^69^, ^70^, ^71^, ^72^ Queries 1–8 concern fundamental photochemistry, are objective, and should elicit direct answers; query 9 concerns a practical matter and should elicit experimental guidance; query 10 is subjective, open‐ended, and could give rise to creative responses; and queries 11–13 concern specific attributes of given substances and should afford commensurably specific responses.

### Q1. Why is the fluorescence spectrum typically the mirror image of the absorption spectrum?

A good response to this query concerning the mirror‐image relationship of absorption and fluorescence spectra should center on molecular vibrational energy levels (in the ground versus excited state) as embodied in the Franck–Condon principle and Kasha's rule, and also express the fact of molecular relaxation in the vibrational manifold of the excited state. The responses from ChatGPT 3.5, Copilot, and Gemini Advanced embodied the Franck–Condon principle and thus were satisfactory, whereas Meta AI described Kasha's rule. The responses from ChatGPT 3.5 and Meta AI were not incorrect but were verbose and lacked salience. The response from ChatGPT 4o was superb: “the same set of vibrational energy levels is involved but in reverse order (excited to ground state for fluorescence and ground to excited state for absorption).”

### Q2. What is the difference between the absorption spectrum and the excitation spectrum?

There are many possible ways to describe the difference between the absorption and the excitation spectrum, and each chatbot provided suitable responses. Among all, Meta AI provided a clear description (albeit grammatically deficient in the use of articles): “Absorption spectrum is a direct measure of absorption, while excitation spectrum is an indirect measure of absorption (via emission).”

### Q3: What is the role of the lowest vibrational level of the excited singlet state in photochemistry?

Excited‐state processes invariably emanate from the lowest vibrational level of the excited singlet state (S_1_) in accord with Kasha's rule. The processes for depopulating the excited singlet state include: (i) fluorescence, by radiative decay from the lowest excited singlet state (S_1_) to the ground state (S_0_); (ii) internal conversion, a non‐radiative process that liberates heat, and also occurs from higher vibrational levels; and (iii) intersystem crossing, a non‐radiative process that gives rise to the excited triplet state (T_1_). All chatbots depicted with remarkable depth the essential aforementioned features. For example, ChatGPT 4o reported under the topic of Fluorescence Emission, “The emission generally occurs from the lowest vibrational level of the excited state, following Kasha's rule, which states that fluorescence occurs predominantly from the lowest excited singlet state regardless of the excitation wavelength.” Copilot reported “In summary, the lowest vibrational level of the excited singlet state is fundamental in photochemistry for ensuring stability, facilitating fluorescence emission, guiding photochemical reactions, determining quantum yields, and defining spectroscopic properties.”

### Q4: Why is the Stokes shift relevant?

The responses given by the chatbots were generally appropriate. ChatGPT 4o first provided a definition and followed with topics: “The Stokes shift is the difference in wavelength (or energy) between the positions of the band maxima of absorption and emission spectra of the same electronic transition. This phenomenon is named after Sir George Stokes, who first described it. The Stokes shift is relevant in several contexts within photochemistry and photophysics for the following reasons:” ChatGPT 4o listed six topics with explanations of each; the topics were “Identification and Characterization of Molecules, Avoiding Reabsorption, Improved Signal Detection, Understanding Molecular Dynamics, Designing Fluorescent Probes, and Energy Transfer Studies.” The response from Meta AI was vague and contained tangential information from the contents of Wikipedia.

### Q5: Why is the excited triplet state generally longer‐lived than the excited singlet state?

The responses given by the chatbots again were generally appropriate. A representative response is illustrated by that from Copilot (with the removal of citations by the authors), “The excited triplet state is generally longer‐lived than the excited singlet state due to the difference in their spin states. When a molecule absorbs a photon, it is excited from its ground state to an excited state. In an excited singlet state, the electron is promoted in the same spin orientation as it was in the ground state (paired). In a triplet excited state, the electron that is promoted has the same spin orientation (parallel) to the other unpaired electron. This difference in spin state makes the transition from singlet to triplet (or triplet to singlet) less likely than the singlet‐to‐singlet transitions. This is because the transition from the triplet state to the singlet ground state involves a change in the spin state of the electron, which is a forbidden transition according to quantum mechanics. This makes it kinetically harder for the excited electron to jump back to the original orbital and at the same time flip its spinning state. As a result, the lifetime of the triplet state is longer than the singlet state by a factor of roughly 10^4^ seconds. Since the triplet state has fewer modes for deactivation (other than to react), it has a relatively long lifetime (on the order of seconds for some molecules). In summary, the longer lifetime of the triplet state compared to the singlet state is due to the difference in their spin states and the quantum mechanical rules that govern their transitions.” Only the response from Meta AI was unclear, even though the description in the provided link was appropriate.

### Q6: Why is (or is not) the lifetime of an excited state important?

The response of Gemini Advanced was concise and accurate: “The lifetime of an excited state is a crucial parameter in photochemistry and photophysics, as it dictates the available time for various processes to occur following the absorption of a photon.” The subsequent text was more expansive: “If an excited state has a short lifetime (picoseconds to nanoseconds), the molecule will quickly relax back to the ground state, predominantly through radiative (fluorescence) or non‐radiative (internal conversion) processes. There's limited time for other processes, such as intersystem crossing to the triplet state or photochemical reactions, to occur.” A subsequent list provided examples of fluorescence‐based techniques (fluorescence lifetime imaging microscopy (FLIM) and Förster resonance energy transfer (FRET)); photochemical reactions (reaction kinetics and product formation); and material properties (photostability, light‐emitting devices). The responses from ChatGPT 3.5 and 4o were equally accurate. The responses from Copilot and Google Advanced were also adequate. On the other hand, the response from Meta AI was unsatisfactory, having drawn on a physics source and Wikipedia, with a tautological closing statement: “The lifetime of an excited state is important as it determines the duration of the excited state and the rate of transition to a lower energy state.”

### Q7: Why does the fluorescence quantum yield of a fluorophore decrease as the fluorescence spectrum shifts to a longer wavelength?

The responses from chatbots should include the concept of the energy gap law, that is, as the fluorescence spectrum shifts to a longer wavelength, the energy gap between the ground and excited state decreases, causing an increase in the rate of non‐radiative decay, which by competition results in diminished fluorescence.^73^, ^74^ Each chatbot (except for Copilot) responded with the term energy gap but with a range of accuracies. ChatGPT 3.5 responded with a completely inaccurate first sentence, “The decrease in fluorescence quantum yield as the fluorescence spectrum shifts to longer wavelengths is primarily due to the phenomenon known as the Stokes shift.” However, some of the subsequent statements were more accurate: “As the emission wavelength shifts to longer wavelengths, the energy of the emitted photons decreases. This decrease in photon energy corresponds to a decrease in the energy gap between the excited state and the ground state of the fluorophore. Consequently, there is a higher probability of vibrational relaxation processes occurring within the excited state, where excess energy is dissipated as heat through molecular vibrations rather than being emitted as fluorescence photons. This non‐radiative decay pathway competes with fluorescence emission, leading to a decrease in fluorescence quantum yield.” ChatGPT 4o lacked the erroneous first sentence, and along with Gemini Advanced and Meta AI, provided satisfactory responses. Copilot provided an irrelevant response that included Planck's Law and the Stokes shift.

### Q8: Why is the quantum yield of phosphorescence generally low for organic compounds in solution?

The present query is related to query 5, which concerns the comparatively long lifetime of the excited triplet state. The ideal responses should include (i) weak spin‐orbit coupling of organic molecules; (ii) flexible molecular motion of organic molecules;^75^, ^76^ and (iii) molecular collisions that underpin intermolecular quenching.^77^ The responses from ChatGPT 3.5 and 4o were accurate and included descriptions of the role of multiple factors. ChatGPT 3.5 reported “Overall, the low quantum yield of phosphorescence for organic compounds in solution is a consequence of spin selection rules, efficient non‐radiative decay pathways, quenching effects, intramolecular vibrational relaxation, and solvent interactions. These factors collectively limit the efficiency of phosphorescence emission in solution environments.” On the other hand, ChatGPT 3.5 appeared off the mark in reporting that “solvents with high viscosity or high polarity can enhance non‐radiative decay pathways and increase the rate of quenching, leading to lower phosphorescence quantum yields.” Gemini Advanced ascribed the causes of low phosphorescence to “several competing processes that can depopulate the triplet excited state (T1) before phosphorescence can occur” and then described such processes. Gemini Advanced alone went further to provide the correct advice that “Embedding the molecule in a rigid matrix, such as a frozen solvent or polymer, restricts molecular motions and reduces collisions, leading to enhanced phosphorescence.” The response from Copilot included keywords such as forbidden transitions, aggregation‐caused quenching, and self‐quenching, but was shorter than those of the other chatbots. The response from Meta AI was an assortment of appropriate looking keywords (e.g., Jablonski diagrams, absorption and emission rates, n → π* excited state) that collectively ranged from meaningless to incorrect. None of the chatbots bit on the subtle feature of the query of “why organic compounds?” as opposed to non‐organic compounds.

### Q9. What is the inner filter effect and how to avoid it?

ChatGPT 4o, Copilot, and Gemini Advanced reported two distinctive inner filter effects (primary: absorption of excitation light by the sample; secondary: reabsorption of fluorescence by the sample) and described practical techniques (e.g., dilution of the sample, front face illumination). The response from Meta AI listed two causes of the secondary inner filter effect: self‐absorption and impurity absorption.

### Q10: Please create the most important photochemistry questions

Chatbots generally responded well to this query, creating meaningful, and in some cases, insightful questions. Many questions generated by chatbots were essentially identical to the queries we fed to chatbots in this section: (i) Franck–Condon principle (related to Q1, by Copilot and Gemini Advanced); (ii) inner filter effects and quenching (Q3, by ChatGPT 4o and Copilot); (iii) non‐radiative decay processes, internal conversion, and intersystem crossing (related to Q4, by ChatGPT 3.5 and 4o); (iv) significance of the Stokes shift (Q5, by ChatGPT 4o, Copilot, and Meta AI); and (v) the differences between singlet and triplet states (related to Q6, by ChatGPT 4o, Gemini Advanced, Copilot, and Meta AI). The strong overlap between the questions we posed (Q1–Q7) and the questions posed by chatbots implies that chatbots are effective in managing at least some conceptual issues central to photochemistry (other scientists would likely pose different types of fundamental questions, given the enormous breadth of the field, and might arrive at different conclusions as to chatbot performance to this subjective query). The following representative questions related to the topics were also generated by chatbots: (i) Jablonski diagram (ChatGPT 4o and Gemini Advanced); (ii) the differences between fluorescence and phosphorescence (ChatGPT 4o, Copilot, Gemini Advanced, and Meta AI); (iii) solvent effects (Gemini Advanced and Meta AI); and (iv) current and future applications of photochemistry (all).

### Q11: Why are azo dyes non‐fluorescent?

Azo dyes are typically non‐fluorescent due to *trans‐cis* photoisomerization and the preference for intersystem crossing leading to non‐radiative decay.^78^, ^79^ The overall responses from ChatGPT 3.5, 4o, and Gemini Advanced were regarded as satisfactory upon description of intersystem crossing and non‐radiative decay. The concept of photoisomerization was covered in depth by ChatGPT 4o and Gemini Advanced. The response from Copilot “a common azo dye, is in the UV region. This means it only absorbs a small amount of visible light, which is not enough to exhibit fluorescence” was utter nonsense; indeed, azo dyes typically absorb reasonably strongly in the visible region and are used extensively in dye industries. A keyword of non‐radiative decay was included in the response from Copilot; however, the response contained the statement “In the case of azo dyes, the excited state molecule can react with oxygen,” which is partially true, but this applies to any molecules. The response from Meta AI was meaningless, with terms such as “electro‐Fenton” and explanations served up as tautological statements: “4. Low fluorescence efficiency: Fluorescent azobenzene derivatives have quite low fluorescence efficiencies.” The responses are summarized in Table 2.

**TABLE 2:** Chatbot responses to Q11.

Chatbots	Rating	Recap of the response
ChatGPT 3.5	Fair	Intersystem crossing
ChatGPT 4o	Good	Intersystem crossing, photoisomerization
Copilot	Poor	(Non‐radiative decay)
Gemini Advanced	Good	Photoisomerization
Meta AI	Poor	Irrelevant (electro‐Fenton)

### Q12: Why do porphyrins typically exhibit two peaks in the fluorescence spectrum?

Porphyrin spectroscopy is quite rich. The absorption spectrum is comprised of a strong band (denoted B, or Soret) in the ultraviolet region and a set of weaker bands (denoted Q) in the visible region, where the number of such visible‐region bands depends on the presence or absence of a chelated metal in the porphyrin macrocycle.^4^, ^80^ The observed fluorescence mirrors the lower energy Q bands and thus reflects coherence with Kasha's rule. The responses are summarized in Table 3. ChatGPT 3.5 reported erroneously that the two bands in the fluorescence spectrum correspond to the Soret and Q bands. ChatGPT 4o denoted the two peaks in the fluorescence spectrum as Q(0,0) and Q(1,0) transitions, which is an acceptable answer albeit located in lengthy text not all of which was correct. Copilot reported the Gouterman four‐orbital model (which indeed is applied to explain porphyrin absorption spectra) but did not address the two‐banded nature of the fluorescence spectrum. Gemini Advanced stated that “the two peaks in the fluorescence spectrum of porphyrins are often referred to as the Q bands (Qx and Qy)” which is incorrect; the two bands do not stem from x‐ and y‐transitions. The response from Meta AI was quite misleading: the response ascribed the cause of two peaks as pH‐dependence, an idea that originates with a study of uroporphyrin and coproporphyrin in aqueous solution over a pH range of 1.1–10.4.^81^ The descriptions in the reference are correct but likely stem from a combination of diverse ionized species and molecular aggregation, and hence are not expected to be of general applicability as proffered by Meta AI. The general failures here of chatbots are (1) the lack of discrimination of the types of porphyrins (free base, metal chelate), and (2) the confusion of two bands in emission with the two major transitions in the absorption spectrum.

**TABLE 3:** Chatbot responses to Q12.

Chatbots	Rating	Recap of the response
ChatGPT 3.5	Wrong	Arose from Soret and Q bands
ChatGPT 4o	Fair	0–0 and 0–1 transitions, Franck–Condon Principle
Copilot	Poor	Gouterman four orbital model
Gemini advanced	Fair	Q_x_ and Q_y_ transitions, 0–0 and 0–1 transitions
Meta AI	Misleading	pH‐dependence

### Q13: Why do Quantum dots typically exhibit high brightness?

Brightness is a product of the molar absorption coefficient at the wavelength of excitation, the fluorescence quantum yield, and the fraction of the fluorescence spectrum that is observed;^82^ thus, high values of both the molar absorption coefficient and the fluorescence quantum yield are beneficial to achieve bright fluorophores. Quantum dots can possess high molar absorption coefficients (up to 1,000,000 M^−1^ cm^−1^)^83^ and a near unity fluorescence quantum yield (~1),^84^ depending on the core size. All of the chatbots, except Meta AI, captured the importance of a high fluorescence quantum yield. The responses are listed in Table 4. The response from ChatGPT 4o and Gemini Advanced mentioned a high molar extinction (absorption) coefficient and a large absorption cross section. The responses from Copilot and Meta AI were wrongly directed due to the presence of keywords (quantum dots and brightness) in the query, which apparently implicated display technologies. Additional prompts such as “in solution” or “in organic solvent” or a definition of brightness may improve the response.

**TABLE 4:** Chatbot responses to Q13.

Chatbots	Rating	Recap of the response
ChatGPT 3.5	Fair	High quantum yield (broad absorption spectra)
ChatGPT 4o	Good	High quantum yield, high molar extinction coefficient
Copilot	Poor	High quantum yield, quantum dot TV
Gemini advanced	Fair	High quantum yield, large absorption cross section
Meta AI	Wrong	Switched the topic to the brightness of QLED, where quantum dots are applied to LCD

## CONCLUSIONS

Four conclusions can be drawn from this study.

1. Chatbots are moderately effective for answering queries concerning fundamental concepts in photochemistry, whereas the responses to more specialized queries for dyes and fluorophores remain mediocre and in some instances are erroneous.
2. Chatbots are complementary to established search engines. The accuracy and reliability of the responses from chatbots remain major concerns; however, the uncertainties likely stem from the data sources employed in the training sets such as web resources, Wikipedia, and data repositories. Incorrect information, errors, and biases are unavoidable even without chatbots. Indeed, modern researchers rely extensively on internet‐based resources (e.g., PubMed abstracts), which are hardly encyclopedic, vetted, and pristine.
3. Chatbots can be seen as the next step in the inexorable advance of language‐centric technology as illustrated along the axis of pencil, typewriter, computer, internet, search engines, and AI. The integration of present AI technologies as manifested by chatbots should not be blindly accepted as a panacea nor apprehensively regarded as a threat. On balance, chatbots appropriately used are expected to facilitate efficiency, freeing scientists to focus on research innovation.
4. Screening of the responses from chatbots by domain experts is absolutely essential. A major limitation of the present use of chatbots is that the correct answer to a query can be embedded in a collection of misstatements and nonsensical text. In other cases, the chatbot's answers are simply wrong. One view might be that the partially if not largely accurate responses from chatbots would comprise a passing grade if posing as a student in a course, and hence should be commended. On the other hand, there is a great difference in expectations for the knowledge of a first‐time learner versus that of a sage, guru, or sensei, which chatbots at present certainly do not reliably match. Only the cognoscenti would know if the chatbot response was correct, completely incorrect, or somewhere in between. Accordingly, unfettered use of the present generation of chatbots in teaching and education warrants significant caution.

## Supporting information


Appendix S1:
