# Supplementary material for: Performance of chatbots in queries concerning fundamental concepts in photochemistry
Source: Photochem Photobiol. 2024 Nov 4;101(4):886–95. doi: 10.1111/php.14037 (PMC12345396; doi:10.1111/php.14037)
Supplement: Supplementary file 1 — Appendix S1: [file PHP-101-886-s001.pdf]

Supporting Information for:

Performance of chatbots in queries concerning fundamental concepts in photochemistry

Masahiko Taniguchi* and Jonathan S. Lindsey

Department of Chemistry, North Carolina State University, Raleigh, NC 27695-8204

Responses to Queries 1–8 (Fundamental questions)

Q1: Why is the fluorescence spectrum typically the mirror image of the absorption spectrum?

ChatGPT 3.5

The relationship between absorption and fluorescence spectra is a consequence of the Jablonski diagram, a conceptual tool used in the study of molecular photochemistry. When a molecule absorbs light, it undergoes a transition from a lower energy state (ground state) to a higher energy state (excited state). This absorption is typically represented by an absorption spectrum, which shows the wavelengths of light that are absorbed by the molecule.

After absorbing light and reaching the excited state, the molecule can return to its ground state by emitting light. This process is known as fluorescence. The emitted light typically has a longer wavelength (lower energy) than the absorbed light, due to energy loss through various relaxation processes within the excited state of the molecule.

The key to understanding why the fluorescence spectrum is often the mirror image of the absorption spectrum lies in the energy levels involved. In absorption, energy is absorbed to promote an electron from a lower energy level to a higher one. In fluorescence, the electron returns to its original, lower energy level, and the excess energy is released as light. Since the energy levels are fixed for a given molecule, the energy difference between the ground state and excited state remains constant.

However, the energy of a photon is inversely proportional to its wavelength ($E = hc/\lambda$, where E is energy, h is Planck's constant, c is the speed of light, and λ is wavelength). Therefore, the longer the wavelength of the emitted light (fluorescence), the lower its energy compared to the absorbed light. This results in a fluorescence spectrum that is typically the mirror image of the absorption spectrum, with peaks at longer wavelengths.

This relationship is not always strictly true due to various factors such as vibrational and rotational relaxation, solvent effects, and interactions with neighboring molecules, but it provides a useful framework for understanding the general behavior of fluorescence and absorption spectra.

ChatGPT 4o
The phenomenon where the fluorescence spectrum appears as the mirror image of the absorption spectrum is primarily due to the nature of electronic transitions and vibrational energy levels in molecules. This can be understood through the principles of the Franck-Condon principle and the Stokes shift. Here's a detailed explanation:
Electronic Transitions and Vibrational Levels
1. **Absorption Process**:
   - When a molecule absorbs light, it is promoted from the ground electronic state (S_0) to an excited electronic state (usually the first excited singlet state, S_1).
   - This transition involves a shift from the ground vibrational level of S_0 to various vibrational levels of S_1.
2. **Fluorescence Process**:
   - After absorption, the molecule typically relaxes to the lowest vibrational level of the excited state S_1 through non-radiative processes (internal conversion and vibrational relaxation).
   - The molecule then returns to the ground electronic state (S_0) by emitting a photon (fluorescence). This transition usually occurs from the lowest vibrational level of S_1 to various vibrational levels of S_0.
Franck-Condon Principle
The Franck-Condon principle states that electronic transitions occur so rapidly that the nuclear configuration of the molecule doesn't change appreciably during the transition. As a result:
- The probability of a transition is highest when there is the greatest overlap between the vibrational wavefunctions of the initial and final states.
- The absorption spectrum reflects transitions from the ground vibrational level of S_0 to multiple vibrational levels of S_1.
- The fluorescence spectrum reflects transitions from the lowest vibrational level of S_1 to multiple vibrational levels of S_0.
Stokes Shift
There is typically a small energy loss between absorption and emission due to non-radiative processes. This causes the emitted fluorescence light to have a slightly longer wavelength (lower energy) compared to the absorbed light. This shift is known as the Stokes shift.
Mirror Image Relationship
Given the Franck-Condon factors:
- The vibrational structure of the excited electronic state mirrors that of the ground state.
- When a molecule absorbs light, the transitions are vertical (in the energy vs. internuclear distance diagram) from the ground state to the excited state.
- When the molecule fluoresces, it transitions from the excited state to the ground state.
Since the same set of vibrational energy levels is involved but in reverse order (excited to ground state for fluorescence and ground to excited state for absorption), the fluorescence spectrum often mirrors the absorption spectrum.
Summary
The mirror image relationship between the fluorescence and absorption spectra arises because both spectra involve transitions between the same sets of vibrational levels, just in reverse order. The absorption spectrum involves transitions from the ground electronic state to the excited state, while the fluorescence spectrum involves transitions from the excited state back to the ground state. The Franck-Condon principle ensures that the most probable transitions (those with the greatest vibrational overlap) are mirrored, resulting in the observed spectral symmetry.

Copilot
The fluorescence spectrum is typically the mirror image of the absorption spectrum due to a principle known as the **Franck-Condon principle** [1]. This principle explains that the electronic transitions in emission spectra are not very different from those of absorption spectra [1].
When a molecule absorbs light, it gets excited from the ground state to a higher energy state. This is represented in the absorption spectrum. When the molecule returns to the ground state, it emits light, which is represented in the fluorescence or emission spectrum [3].
The reason these two spectra are mirror images of each other is due to the equal distribution between the vibrational energy levels of the excited and ground states [3]. The energy differences between the bands in the emission spectrum will be similar to those in the absorption spectrum [2].
However, the emission spectrum is often shifted slightly to the right (longer wavelengths, lower energy) compared to the absorption spectrum. This shift, known as the **Stokes shift**, is due to non-radiative decays (such as internal conversion or vibrational relaxation) that cause the molecule to lose some energy before emitting light [5].
In summary, the mirror-image relationship between absorption and emission spectra is a result of the similar energy transitions involved in both processes, with the slight shift in the emission spectrum accounting for energy lost to non-radiative processes.
Source: Conversation with Copilot, 5/26/2024
Why are the absorption and emission spectra mirror images of each other?
Fluorescence Spectroscopy | JASCO
An Introduction to Fluorescence Spectroscopy - University of California ...
Fluorescence Spectroscopy 1.0 Emission – the mirror image relationship
The Mirror Image Symmetry in the Optical Absorption ... - Springer

Gemini Advanced
The fluorescence spectrum of a molecule often appears as a mirror image of its absorption spectrum due to a few key factors:
1. **Franck-Condon Principle:** This principle states that electronic transitions happen much faster than nuclear motions.
https://chem.libretexts.org/Courses/Pacific_Union_College/Quantum_Chemistry/13%3A_Molecular_Spectroscopy/13.07%3A_The_Franck-Condon_Principle
Therefore, during absorption and emission, the nuclei remain mostly stationary while the electron changes energy levels. This results in vertical transitions in energy diagrams.
2. **Vibrational Energy Levels:** Both ground and excited electronic states have associated vibrational energy levels. The probability of a transition between specific vibrational levels depends on the overlap of their wavefunctions (Franck-Condon factors). These factors are similar for both absorption and emission, leading to a mirror-image relationship in the spectra.
https://en.wikipedia.org/wiki/Franck%E2%80%93Condon_principle
3. **Similar Energy Spacing:** For many fluorophores, the spacing between vibrational energy levels is similar in both the ground and excited states. This means the same transitions are favored for absorption and emission, reinforcing the mirror-image effect.
4. **Stokes Shift:** The emission spectrum is typically shifted to lower energies (longer wavelengths) compared to the absorption spectrum. This is due to energy loss through vibrational

relaxation in the excited state before fluorescence occurs. However, the overall shape of the spectra remains similar.
Exceptions to the Mirror Image Rule:
* **Rigid vs. Flexible Molecules:** The mirror image relationship is more prominent in rigid molecules (e.g., anthracene) where the geometry doesn't change significantly upon excitation. Flexible molecules may exhibit less symmetrical spectra due to changes in geometry.
* **Solvent Effects:** Solvent interactions can influence the vibrational energy levels and distort the mirror-image relationship.
Overall, the mirror-image relationship between absorption and fluorescence spectra is a useful phenomenon for understanding the electronic and vibrational structure of molecules. However, it's essential to consider factors that can cause deviations from this ideal behavior.

Meta AI
The fluorescence spectrum is typically the mirror image of the absorption spectrum due to the principles of spectroscopy and the behavior of molecules during excitation and relaxation processes. Here's a brief explanation:
1. Absorption: When a molecule absorbs light, it transitions from a lower energy state (ground state) to a higher energy state (excited state). The energy of the absorbed photon corresponds to the energy difference between these states.
2. Excited state relaxation: After absorption, the molecule rapidly relaxes to the lowest vibrational level of the excited state through non-radiative processes (e.g., collisions with solvent molecules).
3. Fluorescence emission: From this relaxed excited state, the molecule can emit a photon as it returns to the ground state. The energy of the emitted photon corresponds to the energy difference between the relaxed excited state and the ground state.
4. Mirror image relationship: Since the absorption and emission processes involve the same energy states, the fluorescence spectrum is typically a mirror image of the absorption spectrum. The peak wavelengths are often similar, but the fluorescence spectrum is usually shifted to longer wavelengths (lower energies) due to the relaxation processes that occur before emission.
This mirror image relationship is known as the **Kasha's rule** or **Vavilov's rule**, named after the scientists who first observed and explained this phenomenon. It's a fundamental concept in spectroscopy and is widely used to analyze molecular properties and behavior.

Q2: What is the difference between the absorption spectrum and the excitation spectrum?

ChatGPT 3.5

The absorption spectrum and the excitation spectrum are related but represent different aspects of a molecule's behavior in response to light.

1. **Absorption Spectrum**: This spectrum represents the wavelengths of light that a molecule absorbs. It shows the amount of light absorbed by a sample at different wavelengths. Absorption spectra are commonly measured by passing a broad spectrum of light through a sample and detecting the intensity of light transmitted through the sample. The resulting spectrum shows peaks where the molecule absorbs light, corresponding to specific energy transitions between molecular energy levels.

2. **Excitation Spectrum**: This spectrum represents the efficiency of a molecule's excitation at different wavelengths of light. Unlike the absorption spectrum, which measures the amount of light absorbed, the excitation spectrum measures the intensity of the emitted signal (e.g., fluorescence or phosphorescence) from the molecule when it is excited by light at different wavelengths. In other words, it shows how efficiently the molecule can be excited by light of different energies. Excitation spectra are typically measured by monitoring the intensity of the emitted signal (e.g., fluorescence) while varying the wavelength of the excitation light.

In summary, while the absorption spectrum shows the wavelengths of light absorbed by a molecule, the excitation spectrum shows the efficiency of excitation of the molecule at different wavelengths, typically detected through the emitted signal such as fluorescence. The excitation spectrum can be influenced by factors such as the quantum yield of the excited state, the efficiency of the transition from the excited state back to the ground state (e.g., through fluorescence or non-radiative processes), and any competing processes that occur upon excitation.

ChatGPT 4o

The absorption spectrum and the excitation spectrum are both used to study the interaction of light with a substance, but they have different focuses and are obtained through different experimental setups.

Absorption Spectrum

- **Definition**: The absorption spectrum measures the wavelengths of light absorbed by a substance.
- **Procedure**: It is obtained by passing a broad range of wavelengths of light through the sample and measuring the amount of light absorbed at each wavelength.
- **Information Provided**: This spectrum provides information about the electronic transitions in the molecules, indicating which wavelengths of light are absorbed and therefore the energy levels of the electronic states.
- **Graph**: Typically, it is plotted as absorbance (or absorbance intensity) versus wavelength (or frequency).

Excitation Spectrum

- **Definition**: The excitation spectrum measures the wavelengths of light that can excite the sample to emit fluorescence.

- **Procedure**: It is obtained by fixing the emission wavelength (the wavelength at which fluorescence is monitored) and then varying the excitation wavelength to see which wavelengths result in fluorescence emission.
- **Information Provided**: This spectrum provides information about the efficiency of excitation of different wavelengths and is essentially a mirror of the absorption spectrum for the fluorescent species.
- **Graph**: Typically, it is plotted as fluorescence intensity versus excitation wavelength.
Key Differences
1. **Measurement Focus**:
  - **Absorption Spectrum**: Focuses on the wavelengths absorbed by the sample.
  - **Excitation Spectrum**: Focuses on the wavelengths that can excite the sample to emit fluorescence.
2. **Experimental Setup**:
  - **Absorption Spectrum**: Involves measuring the decrease in intensity of light passing through the sample.
  - **Excitation Spectrum**: Involves measuring the intensity of fluorescence emission while varying the excitation wavelength.
3. **Data Representation**:
  - **Absorption Spectrum**: Absorbance or absorbance intensity vs. wavelength.
  - **Excitation Spectrum**: Fluorescence intensity vs. excitation wavelength.
4. **Purpose**:
  - **Absorption Spectrum**: Provides information about the energy levels of electronic states and the absorption characteristics of the sample.
  - **Excitation Spectrum**: Provides information about the wavelengths that can effectively excite the sample to emit fluorescence, often used to determine the optimal excitation wavelength for fluorescence studies.

In summary, while both spectra provide insights into the interaction of light with a sample, the absorption spectrum focuses on what wavelengths are absorbed, and the excitation spectrum focuses on what wavelengths can excite the sample to produce fluorescence.

Copilot

The **absorption spectrum** and the **excitation spectrum** are both important in understanding how a molecule interacts with light, but they represent different processes:

1. Absorption Spectrum: This shows all of the wavelengths at which light is absorbed by the sample [1]. When light shines on a sample, the sample absorbs certain wavelengths of light, which raises the energy levels of its electrons. This process is measured using a UV-Vis spectrophotometer [1].

2, Excitation Spectrum: This shows the wavelengths of light that a sample will absorb to be able to emit at a specified wavelength [1]. It is essentially a plot of the intensity of fluorescence as a function of the excitation wavelength, while the emission wavelength is kept constant [2]. This process is measured using a fluorescence spectrophotometer [1].

Generally, both the absorption and excitation spectra of a sample will peak at the same wavelength, although there are exceptions [1]. If the sample obeys Kasha’s Rule and Vavilov’s Rule then the excitation spectrum and absorption spectrum will be identical [2]. Excitation spectra can therefore be thought of as fluorescence detected absorption spectra [2].

In summary, while both spectra involve the absorption of light, the absorption spectrum measures all absorbed wavelengths, while the excitation spectrum measures the absorbed wavelengths that lead to fluorescence at a specific emission wavelength.
Source: Conversation with Copilot, 5/26/2024
What is the difference between absorption and excitation spectra?
What are Absorption, Excitation and Emission Spectra?
Difference between an absorbance, emission, and excitation spectrum for ...
Absorption vs. Excitation - What's the Difference? | This vs. That

Gemini Advanced
Both absorption and excitation spectra provide information about the wavelengths of light a molecule absorbs. However, they are obtained through different methods and serve slightly different purposes.
https://www.aatbio.com/resources/faq-frequently-asked-questions/What-is-the-difference-between-absorption-and-excitation-spectra
Absorption Spectrum:
* **How it's measured:** Using a UV-Vis spectrophotometer, the absorbance of a sample is measured across a range of wavelengths. The resulting spectrum shows the wavelengths at which the sample absorbs light and their relative intensities.
https://academic.oup.com/book/41698/chapter/353943288
* **Purpose:** The absorption spectrum provides information about the electronic transitions within a molecule, giving insights into its structure and energy levels.
https://chem.libretexts.org/Bookshelves/Physical_and_Theoretical_Chemistry_Textbook_Maps/Supplemental_Modules_(Physical_and_Theoretical_Chemistry)/Spectroscopy/Electronic_Spectroscopy/Electronic_Spectroscopy_-_Interpretation
Excitation Spectrum:
* **How it's measured:** Using a fluorescence spectrophotometer, the emission intensity of a sample is measured at a fixed wavelength while varying the excitation wavelength. The resulting spectrum shows the wavelengths of light that most effectively excite the sample to produce fluorescence.
* **Purpose:** The excitation spectrum helps identify the optimal excitation wavelength for fluorescence experiments and can provide information about the energy transfer processes within a molecule.
Key Differences:
* **Instrumentation:** Absorption spectra are measured with UV-Vis spectrophotometers, while excitation spectra are measured with fluorescence spectrophotometers.
* **Focus:** Absorption spectra focus on all wavelengths absorbed by the sample, while excitation spectra focus on the wavelengths that lead to fluorescence emission.
https://www.horiba.com/pol/scientific/technologies/fluorescence-spectroscopy/what-is-fluorescence-spectroscopy/
* **Information Gained:** Absorption spectra reveal electronic transitions, while excitation spectra reveal the most efficient wavelengths for inducing fluorescence and can provide insights into energy transfer mechanisms.
Similarity:
* **Peak Overlap:** In many cases, the absorption and excitation spectra of a fluorophore will have similar peak wavelengths, as the wavelengths that a molecule absorbs most efficiently are

often the ones that most effectively lead to fluorescence emission. However, there can be exceptions to this rule, especially in cases where energy transfer processes occur within a molecule.

Meta AI

The absorption spectrum and excitation spectrum are related but distinct concepts in spectroscopy:

Absorption Spectrum:

*Measures the absorption of light by a sample as a function of wavelength (or frequency)

*Represents the probability of a molecule absorbing a photon at a specific energy (wavelength)

*Typically measured by shining light through a sample and detecting the transmitted light

*Peaks in the absorption spectrum correspond to energies where the molecule is most likely to absorb photons

Excitation Spectrum:

*Measures the fluorescence or phosphorescence emission intensity as a function of the excitation wavelength (or frequency)

*Represents the probability of a molecule being excited by a photon at a specific energy (wavelength) and subsequently emitting a photon

*Typically measured by detecting the emitted light while scanning the excitation wavelength

*Peaks in the excitation spectrum correspond to energies where the molecule is most likely to be excited and emit a photon

Key differences:

*Absorption spectrum measures absorption probability, while excitation spectrum measures emission intensity

Absorption spectrum is a direct measure of absorption, while excitation spectrum is an indirect measure of absorption (via emission)

*Excitation spectrum can be influenced by additional factors like fluorescence quantum yield, while absorption spectrum is a more direct measure of molecular properties

In summary, the absorption spectrum shows where a molecule absorbs light, while the excitation spectrum shows where a molecule is most likely to be excited and emit light.

Q3: What is the role of the lowest vibrational level of the excited singlet state in photochemistry?

ChatGPT 3.5

The lowest vibrational level of the excited singlet state plays several important roles in photochemistry:

1. **Radiative Emission**: In many cases, when a molecule is excited to the singlet excited state, it can relax back to the ground state through radiative emission, emitting a photon of light. The lowest vibrational level of the excited singlet state often corresponds to the most energetically favorable transition back to the ground state, leading to the emission of fluorescence or phosphorescence.
2. **Non-Radiative Processes**: The lowest vibrational level of the excited singlet state can also serve as a gateway to various non-radiative relaxation pathways. These pathways involve the dissipation of excess energy through processes such as internal conversion, where the molecule transitions to a lower electronic state of the same spin multiplicity without emitting a photon, or vibrational relaxation, where excess energy is transferred to molecular vibrations and eventually dissipated as heat.
3. **Chemical Reactions**: In some cases, the lowest vibrational level of the excited singlet state can facilitate chemical reactions. For example, photochemical reactions may occur when a molecule in the excited singlet state undergoes reactions that would be energetically unfavorable in the ground state. These reactions can lead to the formation of new chemical species or the rearrangement of existing ones.
4. **Energy Transfer**: The lowest vibrational level of the excited singlet state can also participate in energy transfer processes. For instance, it may transfer its energy to other molecules through processes such as fluorescence resonance energy transfer (FRET) or through collisional quenching, where the excited molecule transfers its energy to a nearby molecule without emitting a photon.

Overall, the lowest vibrational level of the excited singlet state serves as a critical intermediate in the photochemical processes of relaxation, emission, energy transfer, and chemical reactions, playing a fundamental role in the behavior of molecules following absorption of light.

ChatGPT 4o

The lowest vibrational level of the excited singlet state plays a crucial role in photochemistry due to the following reasons:

1. **Stability and Relaxation**: When a molecule absorbs a photon and transitions to an excited singlet state (S1), it often ends up in a higher vibrational level of that excited state. However, it quickly relaxes to the lowest vibrational level of the excited state through non-radiative processes such as internal conversion and vibrational relaxation. This rapid relaxation ensures that the molecule reaches a stable excited state before undergoing further photochemical reactions or emission.
2. **Fluorescence Emission**: The lowest vibrational level of the excited singlet state is typically the starting point for fluorescence emission. When the molecule returns to the ground state (S0), it often emits a photon, resulting in fluorescence. The emission generally occurs from the lowest

vibrational level of the excited state, following Kasha's rule, which states that fluorescence occurs predominantly from the lowest excited singlet state regardless of the excitation wavelength.
3. **Photochemical Reactions**: The photochemical behavior of a molecule, including reactions such as isomerization, electron transfer, or energy transfer, often depends on the molecule being in the lowest vibrational level of the excited singlet state. This state provides a consistent and well-defined energy level from which these reactions can proceed, leading to predictable photochemical pathways.
4. **Quantum Yield and Efficiency**: The quantum yield of photochemical processes, including fluorescence, phosphorescence, and photochemical reactions, is influenced by the efficiency with which a molecule reaches and remains in the lowest vibrational level of the excited singlet state. Efficient relaxation to this state increases the likelihood of specific photochemical outcomes and can enhance the overall efficiency of the processes.
5. **Spectroscopic Characteristics**: The spectral properties of a molecule, such as the positions and intensities of absorption and emission peaks, are influenced by the transitions involving the lowest vibrational level of the excited singlet state. These transitions determine the characteristic absorption and fluorescence spectra of the molecule, which are essential for spectroscopic analysis and identification.
In summary, the lowest vibrational level of the excited singlet state is fundamental in photochemistry for ensuring stability, facilitating fluorescence emission, guiding photochemical reactions, determining quantum yields, and defining spectroscopic properties.

Copilot
The lowest vibrational level of the excited singlet state, often denoted as S1, plays a crucial role in photochemistry [4].
When a molecule absorbs a photon, it is excited from its ground state (S0) to an excited state (Sn). This excited state is usually a higher vibrational level of the excited singlet state [2]. The molecule then undergoes vibrational relaxation, a process where it loses energy through vibrations and transitions to the lowest vibrational level of the excited singlet state (S1) [5].
The S1 state is significant because fluorescence emission, a key process in photochemistry, occurs from this state [4]. When a photon is released from the fluorochrome (the molecule that is fluorescing), the molecule transitions from the S1 state back to one of the several vibrational levels of the electronic ground state [4]. This photon emission is what we observe as fluorescence [4].
Moreover, the S1 state is also important in non-radiative processes. For instance, internal conversion, a process where the molecule transitions from the S1 state to the ground state without emitting a photon, also occurs from the S1 state [5].
In summary, the lowest vibrational level of the excited singlet state (S1) is crucial in photochemistry as it is the state from which most radiative and non-radiative processes occur.
Source: Conversation with Copilot, 5/26/2024
Fluorescence: General Aspects | SpringerLink
Principles of Photochemical Reactions | SpringerLink
Kasha's rule - Wikipedia
Fundamentals of Photochemistry: Excited State Formation ... - Springer
Photochemistry - Michigan State University

Gemini Advanced

The lowest vibrational level (v'=0) of the excited singlet state (S1) plays a crucial role in photochemistry due to several reasons:

1. **Fluorescence:** Fluorescence is the emission of a photon when a molecule relaxes from the lowest vibrational level of the excited singlet state (S1, v'=0) to the ground state (S0).
https://www.olympus-lifescience.com/es/microscope-resource/primer/techniques/confocal/fluoroexciteemit/
This is the most common radiative decay pathway for excited singlet states.
2. **Internal Conversion:** Internal conversion is a non-radiative process where the molecule transitions from a higher vibrational level of S1 to the lowest vibrational level (S1, v'=0) without emitting a photon.
https://en.wikipedia.org/wiki/Internal_conversion_(chemistry)
This process is usually very fast and efficient, leading to a majority of excited molecules populating the v'=0 level before further relaxation.
3. **Intersystem Crossing:** Intersystem crossing is another non-radiative process where a molecule transitions from the singlet excited state (S1, v'=0) to a triplet excited state (T1). This process is typically slower than internal conversion and depends on the spin-orbit coupling in the molecule.
4. **Photochemical Reactions:** The lowest vibrational level of the excited singlet state is often the starting point for photochemical reactions. The excess energy from the absorbed photon can be used to overcome activation barriers for various chemical transformations, such as isomerizations, bond cleavages, and cycloadditions.
5. **Kasha's Rule:** Kasha's rule states that fluorescence (and phosphorescence) usually occurs from the lowest vibrational level of the excited state.
https://www.edinst.com/blog/what-is-kashas-rule/
This is because internal conversion rapidly depopulates higher vibrational levels, making them less likely to emit photons.

In summary, the lowest vibrational level of the excited singlet state acts as a critical intermediate in photochemistry. It serves as the starting point for fluorescence, internal conversion, intersystem crossing, and various photochemical reactions. Understanding the dynamics and reactivity of this state is essential for predicting and controlling photochemical processes.

Meta AI

The role of the lowest vibrational level of the excited singlet state in photochemistry includes [1,2]:

1. Energy Transfer: The lowest vibrational level of the excited singlet state can transfer energy to other molecules, leading to photosensitization and the formation of a triplet excited state.
2. Internal Conversion: The excited molecule can undergo internal conversion, where it transitions to a lower vibrational level of the excited singlet state, releasing excess energy as heat.
3. Intersystem Crossing: The excited molecule can undergo intersystem crossing, where it transitions to a triplet excited state, which can lead to phosphorescence.
4. Vibrational Relaxation: The excited molecule can undergo vibrational relaxation, where it loses excess vibrational energy and transitions to the lowest vibrational level of the excited singlet state.
5. Fluorescence: The excited molecule can emit a photon and return to the ground state, resulting in fluorescence.

In summary, the lowest vibrational level of the excited singlet state plays a crucial role in photochemistry as it can lead to various energy transfer processes, including internal conversion, intersystem crossing, vibrational relaxation, and fluorescence. These processes are important in understanding photochemical reactions and their applications in fields such as photomedicine and optoelectronics.
https://employees.csbsju.edu/cschaller/Reactivity/photochem/PCfluorescence.htm
https://chem.libretexts.org/Bookshelves/Physical_and_Theoretical_Chemistry_Textbook_Maps/Map%3A_Physical_Chemistry_for_the_Biosciences_(Chang)/14%3A_Spectroscopy/14.07%3A_Fluorescence_and_Phosphorescence

Q4: Why is the Stokes shift relevant?

ChatGPT 3.5

The Stokes shift is relevant in various fields, especially in spectroscopy, photochemistry, and fluorescence imaging. Here's why:

1. **Spectral Discrimination**: The Stokes shift provides a convenient way to distinguish between excitation and emission wavelengths in spectroscopic measurements. Because the emitted light (fluorescence or phosphorescence) typically occurs at longer wavelengths than the absorbed light (excitation), the Stokes shift allows researchers to selectively detect the emitted signal without interference from the excitation light.
2. **Reducing Background Interference**: By separating the excitation and emission wavelengths, the Stokes shift helps minimize background interference in fluorescence measurements. This interference can arise from scattered excitation light or from other fluorophores or chromophores present in the sample. The Stokes shift ensures that the emitted fluorescence signal is distinct from any residual excitation light, improving the sensitivity and specificity of fluorescence measurements.
3. **Energy Dissipation**: The energy difference between the excitation and emission wavelengths, represented by the Stokes shift, reflects the energy lost during the relaxation process from the excited state to the ground state. This energy dissipation occurs through various relaxation mechanisms, such as vibrational relaxation, rotational relaxation, and solvent relaxation. Understanding the Stokes shift provides insights into the dynamics of these relaxation processes and the energetics of excited state transitions.
4. **Molecular Structure and Environment**: The magnitude of the Stokes shift can be influenced by the molecular structure of the fluorophore or chromophore, as well as the surrounding environment (e.g., solvent polarity, temperature, pH). Changes in the molecular environment can alter the energy levels and relaxation pathways, leading to variations in the Stokes shift. Therefore, monitoring the Stokes shift can provide information about the molecular structure, dynamics, and local environment of the fluorophore or chromophore.
5. **Applications**: The Stokes shift is critical in various applications, including fluorescence spectroscopy, fluorescence microscopy, fluorescence imaging, chemical sensing, and molecular diagnostics. By exploiting the Stokes shift, researchers can design sensitive and selective assays, probe molecular interactions, track biological processes, and visualize cellular structures and dynamics with high spatial and temporal resolution.

Overall, the Stokes shift is a fundamental concept in spectroscopy and fluorescence science, providing valuable information about the energy dissipation, molecular properties, and applications of fluorescent and chromophoric molecules.

ChatGPT 4o

The Stokes shift is the difference in wavelength (or energy) between the positions of the band maxima of absorption and emission spectra of the same electronic transition. This phenomenon is named after Sir George Stokes, who first described it. The Stokes shift is relevant in several contexts within photochemistry and photophysics for the following reasons:

1. **Identification and Characterization of Molecules**: The Stokes shift is a characteristic property of a molecule and can be used to identify and distinguish between different species. Each molecule has a unique Stokes shift that can serve as a molecular fingerprint in fluorescence spectroscopy.
2. **Avoiding Reabsorption**: In practical applications, such as in fluorescence microscopy or sensors, a large Stokes shift helps to separate the excitation light from the emitted fluorescence. This separation minimizes reabsorption of the emitted light by the sample, leading to clearer and more accurate fluorescence measurements.
3. **Improved Signal Detection**: A significant Stokes shift enhances the sensitivity and specificity of detection systems by reducing background noise. By ensuring that the emitted light is at a different wavelength than the excitation light, detectors can more easily distinguish between the two, improving the signal-to-noise ratio.
4. **Understanding Molecular Dynamics**: The magnitude of the Stokes shift provides insights into the molecular environment and the dynamics of the excited state. A large Stokes shift often indicates significant changes in the molecular structure or environment between the ground and excited states, such as solvent relaxation, conformational changes, or interactions with the surrounding matrix.
5. **Designing Fluorescent Probes**: In the design of fluorescent dyes and probes, a large Stokes shift is desirable to minimize overlap between absorption and emission spectra. This helps in developing more effective and versatile fluorescent labels for biological and chemical sensing applications.
6. **Energy Transfer Studies**: The Stokes shift is crucial in studies involving energy transfer mechanisms, such as Förster Resonance Energy Transfer (FRET). Understanding the Stokes shift allows researchers to select appropriate donor-acceptor pairs with minimal spectral overlap, optimizing the efficiency of energy transfer and the accuracy of distance measurements at the molecular level.
In summary, the Stokes shift is a fundamental parameter that affects the interpretation of fluorescence data, the design of fluorescence-based technologies, and the understanding of photophysical and photochemical processes in various molecular systems.

Copilot
The Stokes shift is highly relevant in fluorescence spectroscopy and photochemistry for several reasons:
1. Separation of Absorption and Emission Spectra: The Stokes shift allows for the separation of the absorption and emission spectra [1]. This is important because it reduces the overlap between the two spectra, which can interfere with the detection of fluorescence [4].
2. Reduction of Fluorescence Quenching: By separating the absorption and emission spectra, the Stokes shift helps to reduce the quenching of fluorescence [4]. Quenching is a process that decreases the fluorescence intensity, so reducing it can lead to a stronger signal [4].
3. Sensitive Reporter of Local Environment: The Stokes shift is sensitive to the local environment of the fluorophore (the molecule that is fluorescing) [1]. Changes in the Stokes shift can provide information about changes in the environment around the fluorophore [1].
4. Facilitates Biological Imaging: In biological imaging, a larger Stokes shift can provide a stronger signal by reducing interference and eliminating quenching of fluorescence [4]. This makes it easier to detect and image biological samples [4].

5. Understanding Molecular Interactions: The Stokes shift can provide insights into molecular interactions, such as solvent-solute interactions [2]. It can indicate how much the solvent molecules reorient or relax around the excited molecule [2].
In summary, the Stokes shift is a key parameter in fluorescence spectroscopy and photochemistry, providing valuable information about molecular behavior and interactions, and facilitating the detection and imaging of fluorescent samples.
Source: Conversation with Copilot, 5/26/2024
10.3.2: The Stokes Shift - Chemistry LibreTexts
Why does the Stokes shift occur? – Sage-Tips
Stokes shift - Wikipedia
18.1: Theory of Raman Spectroscopy - Chemistry LibreTexts
The term fluorescence was coined by Stokes circa 1850 to name a ...
Stokes shift | physics | Britannica
en.wikipedia.org

Gemini Advanced
The Stokes shift is a crucial concept in fluorescence spectroscopy with numerous applications in various fields:
1. **Distinguishing Fluorescence from Scattered Light:** The Stokes shift allows us to differentiate fluorescence from scattered light (Rayleigh or Raman scattering) which have the same wavelength as the excitation light. This is essential for accurate fluorescence measurements.
2. **Probing Molecular Environments:** The magnitude of the Stokes shift depends on the environment surrounding the fluorophore.
https://www.edinst.com/blog/what-is-the-stokes-shift/
Changes in polarity, viscosity, or specific interactions with other molecules can alter the energy levels of the fluorophore, leading to changes in the Stokes shift.
https://chem.libretexts.org/Courses/Providence_College/CHM_331_Advanced_Analytical_Chemistry_1/10%3A_Molecular_Luminescence_Spectrometry/10.03%3A_Applications_of_Photoluminescence_Methods/10.3.02%3A_The_Stokes_Shift
This property is used to study protein folding, membrane dynamics, and other biological processes.
3. **Fluorescence Microscopy and Imaging:** In fluorescence microscopy, the Stokes shift allows the separation of excitation and emission light using filters.
https://www.aatbio.com/resources/faq-frequently-asked-questions/What-is-Stokes-Shift
This enables the visualization of fluorescently labeled molecules against a dark background, enhancing contrast and sensitivity.
4. **FRET (Förster Resonance Energy Transfer):** FRET is a process where energy is transferred from a donor fluorophore to an acceptor fluorophore through dipole-dipole interactions.
https://www.olympus-lifescience.com/en/microscope-resource/primer/techniques/fluorescence/fret/fretintro/
The Stokes shift of the donor and acceptor molecules is crucial for efficient FRET. It allows the donor emission to overlap with the acceptor absorption, enabling energy transfer.
5. **Material Science and Nanotechnology:** Stokes shift is used to characterize the properties of fluorescent materials, such as quantum dots and nanoparticles. It provides insights into their electronic structure and interactions with their environment.

6. **Environmental Monitoring:** Fluorescent probes with specific Stokes shifts can be used to detect and quantify pollutants or toxins in environmental samples. The change in Stokes shift upon binding to a target molecule can be used as a signal for detection.
In summary, the Stokes shift is a fundamental concept in fluorescence with widespread applications in various fields due to its ability to separate fluorescence from scattered light, probe molecular environments, and enable various fluorescence-based techniques.

Meta AI
The Stokes shift is relevant for several reasons [1 2]:
1. Raman spectroscopy: Analyzing the intensity and frequency of the spectral shift in Raman spectroscopy provides valuable information about the vibrational modes of molecules, enabling the identification of chemical bonds, functional groups, and molecular conformations.
2. Yttrium oxysulfide: Yttrium oxysulfide doped with gadolinium oxysulfide is a common industrial anti-Stokes pigment, absorbing in the near-infrared and emitting in the visible region of the spectrum, making it valuable in security printing, anti-counterfeiting measures, and luminescent displays.
3. Photon upconversion: Photon upconversion is an anti-Stokes process where lower-energy photons are converted into higher-energy photons, observed in Raman spectroscopy and upconverting nanoparticles, enabling the determination of a material's temperature.
4. Optoelectronic devices: Stokes shifted emission in direct-bandgap thin-film semiconducting layers originates from doping, strain, and disorder, making controlling these factors crucial for optimizing device performance in optoelectronic devices.
5. Fluorescence methods: The Stokes shift is critical to the sensitivity of fluorescence methods as it enables a good detection of photons against a low background as well as the separation from an excitation source.
6. Biological imaging: Large Stokes shifts are helpful for in vitro biological imaging, allowing for the detection of photons against a low background and separation from an excitation source.
7. Chemical sensors: Push–pull compounds with ICT characteristics are widely used as chemical sensors, and their Stokes shift is dependent on environmental parameters like solvent polarity or viscosity.
8. Non-linear materials: Compounds with large Stokes shifts have attracted attention for their possible applications in photoelectronic devices, as non-linear materials, emitters, or in organic light-emitting diodes (OLED).
The Stokes shift is a crucial concept in understanding various spectroscopic phenomena and has significant applications in fields like Raman spectroscopy, optoelectronic devices, biological imaging, and chemical sensors. Its relevance lies in providing valuable information about molecular structures, enabling the identification of chemical bonds and functional groups, and playing a critical role in the sensitivity of fluorescence methods.
https://en.wikipedia.org/wiki/Stokes_shift
https://www.mdpi.com/1420-3049/25/11/2488

Q5: Why is the excited triplet state generally longer-lived than the excited singlet state?

ChatGPT 3.5

The excited triplet state is generally longer-lived than the excited singlet state due to several factors related to the quantum mechanical selection rules and the nature of the electronic states involved:

1. **Spin Multiplicity**: The singlet and triplet states differ in their spin multiplicity. Singlet states have total spin quantum number (S) equal to zero, while triplet states have S equal to one. The transition between singlet and triplet states violates the conservation of spin angular momentum, making it a spin-forbidden process. This spin-forbiddenness generally results in a longer lifetime for the triplet state compared to the singlet state.
2. **Radiative and Non-Radiative Decay Pathways**: In many molecules, the radiative decay pathway from the excited singlet state (singlet-singlet transition) is more efficient compared to the radiative decay pathway from the excited triplet state (triplet-triplet transition). This is because the radiative transition from the triplet state to the ground state (phosphorescence) is spin-forbidden and therefore less likely to occur. Consequently, the excited triplet state often undergoes non-radiative decay processes, such as intersystem crossing (ISC) and internal conversion (IC), which involve transitions to lower energy electronic states of the same spin multiplicity. These non-radiative processes can significantly extend the lifetime of the triplet state.
3. **Kinetic Accessibility of Decay Pathways**: The efficiency of radiative and non-radiative decay pathways depends on the kinetic accessibility of these processes. In many cases, the triplet state is kinetically trapped, meaning that the pathways leading to its decay are relatively slow compared to those for the singlet state. This kinetic trapping can result from factors such as the geometry of the molecule, the presence of heavy atoms, or the nature of the surrounding environment.
4. **Energy Gap**: The energy gap between the excited singlet and triplet states can also influence their lifetimes. Generally, the triplet state is lower in energy than the singlet state, leading to a larger energy gap between the triplet excited state and the ground state. This larger energy gap can reduce the rate of spontaneous emission from the triplet state, contributing to its longer lifetime.

Overall, the longer lifetime of the excited triplet state compared to the excited singlet state is a consequence of spin selection rules, the efficiency of radiative and non-radiative decay pathways, the kinetic accessibility of these pathways, and the energy gap between electronic states.

ChatGPT 4o

The excited triplet state is generally longer-lived than the excited singlet state due to differences in spin multiplicity and the rules governing electronic transitions in molecules. Here are the key reasons:

1. **Spin Multiplicity and Selection Rules**:
   - In a singlet state, all electrons in the molecule are paired, meaning their spins are opposite (antiparallel).
   - In a triplet state, two electrons have parallel spins. This difference in spin configuration affects the transition probabilities between states.

- The transition from an excited singlet state to the ground singlet state (a singlet-to-singlet transition) is spin-allowed, meaning it follows the selection rules that govern electronic transitions. Such transitions are generally fast and efficient.
- In contrast, the transition from an excited triplet state to the ground singlet state (a triplet-to-singlet transition) is spin-forbidden, meaning it violates the spin selection rules. As a result, this transition occurs much less frequently and is significantly slower.
2. **Intersystem Crossing and Phosphorescence**:
- When a molecule in an excited singlet state undergoes intersystem crossing, it can transition to an excited triplet state. This process is relatively slow compared to internal conversion (a non-radiative transition within states of the same multiplicity), but once in the triplet state, the molecule remains there for a longer time due to the spin-forbidden nature of the triplet-to-singlet transition.
- The emission of light from the triplet state to the ground state is known as phosphorescence. Because the triplet-to-singlet transition is spin-forbidden, phosphorescence has a much longer lifetime compared to fluorescence (which is the emission from the singlet excited state to the ground state).
3. **Energy Dissipation**:
- In the triplet state, molecules can lose energy through non-radiative processes such as collisions with other molecules or through interactions with the environment. However, these processes are typically slower compared to the radiative and non-radiative processes in the singlet state due to the spin-forbidden nature of the transitions.
4. **Environmental Factors**:
- The lifetime of the triplet state can be influenced by the molecular environment, including factors such as the presence of oxygen, which can quench the triplet state and shorten its lifetime. However, in the absence of such quenching agents, the triplet state remains longer-lived due to the intrinsic properties of spin-forbidden transitions.

In summary, the longer lifetime of the excited triplet state compared to the excited singlet state is primarily due to the spin-forbidden nature of the triplet-to-singlet transition, which results in much slower radiative and non-radiative decay processes.

Copilot

The excited triplet state is generally longer-lived than the excited singlet state due to the difference in their spin states [1] [2] [4].

When a molecule absorbs a photon, it is excited from its ground state to an excited state. In an excited singlet state, the electron is promoted in the same spin orientation as it was in the ground state (paired). In a triplet excited state, the electron that is promoted has the same spin orientation (parallel) to the other unpaired electron [1] [2].

This difference in spin state makes the transition from singlet to triplet (or triplet to singlet) less likely than the singlet-to-singlet transitions [1] [2]. This is because the transition from the triplet state to the singlet ground state involves a change in the spin state of the electron, which is a forbidden transition according to quantum mechanics [1] [2]. This makes it kinetically harder for the excited electron to jump back to the original orbital and at the same time flip its spinning state [1].

As a result, the lifetime of the triplet state is longer than the singlet state by a factor of roughly
10^410^4
seconds [4]. Since the triplet state has fewer modes for deactivation (other than to react), it has a relatively long lifetime (on the order of seconds for some molecules) [3].

In summary, the longer lifetime of the triplet state compared to the singlet state is due to the difference in their spin states and the quantum mechanical rules that govern their transitions.
Source: Conversation with Copilot, 5/26/2024
photochemistry - Is a triplet state or singlet state more stable ...
14.7: Fluorescence and Phosphorescence - Chemistry LibreTexts
15.1: Electronically Excited Molecules - Chemistry LibreTexts
The Triplet State - Springer

Gemini Advanced
The excited triplet state (T1) is generally longer-lived than the excited singlet state (S1) due to the nature of the electronic transitions involved in their relaxation back to the ground state (S0).
https://chem.libretexts.org/Courses/BethuneCookman_University/BCU%3A_CH_332_Physical_Chemistry_II/Text/15%3A_Lasers_Laser_Spectroscopy_and_Photochemistry/15.1%3A_Electronically_Excited_Molecules
Spin Multiplicity and Selection Rules:
* **Singlet state (S1):** In the excited singlet state, all electrons in the molecule are paired with opposite spins.
https://chem.libretexts.org/Courses/BethuneCookman_University/BCU%3A_CH_332_Physical_Chemistry_II/Text/15%3A_Lasers_Laser_Spectroscopy_and_Photochemistry/15.1%3A_Electronically_Excited_Molecules
The transition back to the ground state S0 (also a singlet state) is spin-allowed, meaning it does not require a change in electron spin. This allows for a rapid transition through fluorescence, typically occurring on the nanosecond timescale.
* **Triplet state (T1):** In the excited triplet state, two electrons have the same spin orientation (parallel spins).
https://chem.libretexts.org/Courses/BethuneCookman_University/BCU%3A_CH_332_Physical_Chemistry_II/Text/15%3A_Lasers_Laser_Spectroscopy_and_Photochemistry/15.1%3A_Electronically_Excited_Molecules
The transition back to the ground state S0 is spin-forbidden, meaning it requires a change in electron spin. This makes the transition less probable and thus slower, leading to a longer lifetime for the triplet state, often in the microsecond to second range.
Intersystem Crossing:
The transition from the excited singlet state (S1) to the excited triplet state (T1) is called intersystem crossing (ISC). This process is also spin-forbidden, but it can occur due to spin-orbit coupling, a weak interaction between the electron's spin and its orbital motion.
https://www.oxfordreference.com/display/10.1093/oi/authority.20110803100008607
The efficiency of ISC depends on the molecular structure and the presence of heavy atoms, which enhance spin-orbit coupling.
https://pubs.acs.org/doi/10.1021/jp010187w
Phosphorescence:
The relaxation of the excited triplet state (T1) to the ground state (S0) can occur through phosphorescence, which is the emission of a photon.
https://chem.libretexts.org/Bookshelves/Physical_and_Theoretical_Chemistry_Textbook_Maps/Supplemental_Modules_(Physical_and_Theoretical_Chemistry)/Spectroscopy/Electronic_Spectroscopy/Radiative_Decay/Phosphorescence

However, due to the spin-forbidden nature of the transition, phosphorescence is much less likely than fluorescence, resulting in the longer lifetime of the triplet state.
Summary:
In essence, the difference in spin multiplicity and the resulting selection rules for the transitions to the ground state lead to the difference in lifetimes between the excited singlet and triplet states. The spin-forbidden nature of the triplet state transition makes it less probable and slower, resulting in a longer-lived state.

Meta AI
The excited triplet state is generally longer-lived than the excited singlet state for several reasons [1]:
1. Spin state: The transition from singlet to triplet (or reverse) involves a change in electronic state, which makes it improbable and leads to a longer lifetime for the triplet state.
2. Radiation: The radiation that induces the transition from the ground to the excited triplet state has a low probability of occurring, resulting in less intense absorption bands.
3. Energy contributions: The energy of the triplet state is lower than that of the singlet state due to the exchange term, which exists only for electron pairs with the same spin.
4. Kinetic factors: The triplet state is kinetically harder for the excited electron to jump back to the original orbital and flip its spinning state, leading to a longer lifetime.
5. Stability and lifetime: Stability refers to the energy level (a thermodynamic factor), while lifetime is a kinetic factor, and they do not necessarily agree with each other.
Please note that the excited triplet state's lifetime varies depending on the specific molecule and spin system.
I hope this helps! Let me know if you have any other questions.
https://chemistry.stackexchange.com/questions/24107/is-a-triplet-state-or-singlet-state-more-stable

Q6: Why is (or is not) the lifetime of an excited state important?

ChatGPT 3.5

The lifetime of an excited state is indeed important and can have significant implications in various fields of science, including chemistry, physics, materials science, and biology. Here's why:

1. **Radiative Processes**: The lifetime of an excited state determines the probability of radiative processes such as fluorescence or phosphorescence. A longer excited state lifetime increases the likelihood of radiative decay, leading to stronger fluorescence or phosphorescence signals. Therefore, the lifetime of an excited state directly influences the brightness and intensity of emitted light, which is crucial in fluorescence-based techniques such as fluorescence spectroscopy, fluorescence microscopy, and fluorescence imaging.
2. **Non-Radiative Processes**: In addition to radiative decay, excited states can undergo non-radiative processes such as internal conversion and intersystem crossing. These processes involve the dissipation of excess energy through interactions with the surrounding environment, leading to relaxation to lower energy states without emission of photons. The lifetime of an excited state affects the competition between radiative and non-radiative pathways, thereby influencing the overall efficiency of light emission and energy transfer processes.
3. **Chemical Reactivity**: The lifetime of an excited state can impact the chemical reactivity of molecules. Excited states with longer lifetimes may have more time to undergo photochemical reactions, such as photoisomerization, photodissociation, or energy transfer reactions. Understanding the lifetime of excited states is crucial for predicting and controlling the outcome of photochemical reactions, which have applications in areas such as photovoltaics, photocatalysis, and photodynamic therapy.
4. **Materials Properties**: Excited state lifetimes play a critical role in determining the optical, electronic, and photophysical properties of materials. For example, in organic light-emitting diodes (OLEDs), the excited state lifetime of luminescent materials affects the device efficiency, lifetime, and color purity. In semiconductor materials, the excited state lifetime influences carrier recombination dynamics, charge transport properties, and photoluminescence behavior.
5. **Biological Applications**: In biological systems, the lifetime of fluorescent probes and biomolecules in excited states provides valuable information about molecular structure, dynamics, interactions, and environmental conditions. Fluorescence lifetime imaging microscopy (FLIM) and time-resolved fluorescence spectroscopy (TRFS) techniques exploit the lifetime of excited states to study biological processes at the molecular and cellular levels, including protein-protein interactions, ion fluxes, and intracellular signaling events.

In summary, the lifetime of an excited state is important because it governs the efficiency of radiative and non-radiative processes, influences chemical reactivity, determines materials properties, and enables a wide range of applications in science and technology. Understanding and controlling excited state lifetimes are therefore crucial for the design and optimization of photonic, electronic, and biological systems.

ChatGPT 4o

The lifetime of an excited state is important for several reasons, depending on the context and applications in which it is being considered. Here are some key reasons why the lifetime of an excited state matters:

1. **Photophysical Studies**: The lifetime of an excited state provides valuable information about the dynamics and mechanisms of molecular processes. By studying the lifetimes, researchers can gain insights into the rates of various radiative and non-radiative transitions, intersystem crossing, internal conversion, and other relaxation processes.

2. **Fluorescence and Phosphorescence Applications**: In fluorescence and phosphorescence spectroscopy and imaging, the excited state lifetime affects the intensity and duration of the emitted light. Longer lifetimes are generally associated with phosphorescence, while shorter lifetimes are characteristic of fluorescence. These properties are critical for applications such as time-resolved fluorescence spectroscopy, fluorescence lifetime imaging microscopy (FLIM), and the design of fluorescent probes and sensors.

3. **Energy Transfer Processes**: The efficiency of energy transfer processes, such as Förster Resonance Energy Transfer (FRET) and Dexter energy transfer, depends on the lifetimes of the donor and acceptor molecules' excited states. The lifetime influences the distance over which energy transfer can occur and the overall efficiency of the process.

4. **Chemical Reactivity**: The lifetime of an excited state can influence the chemical reactivity of a molecule. Molecules in excited states often have different reactivity compared to their ground state counterparts. Longer lifetimes provide a greater window of time for chemical reactions to occur, enabling photochemical and photophysical processes that would not be feasible with shorter-lived excited states.

5. **Material Properties**: In materials science, the excited state lifetime can impact the performance of materials such as organic light-emitting diodes (OLEDs), photovoltaic cells, and photocatalysts. Longer-lived excited states can enhance the efficiency of light emission, charge separation, and energy conversion processes.

6. **Biological Systems**: In biological systems, the excited state lifetime of chromophores and fluorophores can affect processes such as photosynthesis, vision, and photoprotection. Understanding these lifetimes helps elucidate the underlying mechanisms of these biological functions and can inform the design of biomimetic systems.=

7. **Environmental Sensitivity**: The lifetime of an excited state can be sensitive to the local environment, such as solvent polarity, viscosity, temperature, and the presence of quenchers. This sensitivity makes excited state lifetimes useful as probes for studying microenvironments and for sensing applications.

In summary, the lifetime of an excited state is important because it provides critical information about the behavior of molecules and materials under excitation, influences the efficiency and mechanisms of various photophysical and photochemical processes, and has practical implications for a wide range of scientific and technological applications.

Copilot
The lifetime of an excited state is indeed important in photochemistry and spectroscopy for several reasons:
1. Determines Emission Processes: The lifetime of an excited state determines whether a molecule will undergo fluorescence (short-lived excited states) or phosphorescence (long-lived excited states) [2]. This is crucial in understanding the photophysical properties of a molecule [2].
2. Influences Reaction Pathways: The longer a molecule stays in an excited state, the more likely it is to undergo chemical reactions [1]. Therefore, the lifetime of the excited state can influence the reaction pathways that a molecule can take [1].
3. Affects Spectroscopic Measurements: The lifetime of the excited state affects the intensity and shape of the emission spectrum in spectroscopic measurements [1]. This is because the intensity of the emitted light is proportional to the time the molecule spends in the excited state [1].
4. Provides Information About Molecular Environment: The lifetime of the excited state can provide information about the molecular environment [1]. For example, changes in the lifetime can indicate changes in the local environment, such as solvent polarity or temperature [1].
5. Used in Time-Resolved Spectroscopy: The lifetime of the excited state is a key parameter in time-resolved spectroscopy [4] [5]. This technique can provide detailed information about the dynamic processes that occur after a molecule absorbs light [4] [5].
In summary, the lifetime of an excited state is important as it influences the emission processes, reaction pathways, spectroscopic measurements, and provides valuable information about the molecular environment and dynamic processes.
Source: Conversation with Copilot, 5/26/2024
Fluorescence and Phosphorescence - Chemistry LibreTexts
3.2: Energy States and Transitions - Chemistry LibreTexts
Excited states - overview | Numerade
Excited states - overview | Numerade
Excited state - Wikipedia

Gemini Advanced
The lifetime of an excited state is a crucial parameter in photochemistry and photophysics, as it dictates the available time for various processes to occur following the absorption of a photon. Here's why it's important:
1. Determining the Fate of Excited Molecules:
* **Short-Lived Excited States:** If an excited state has a short lifetime (picoseconds to nanoseconds), the molecule will quickly relax back to the ground state, predominantly through radiative (fluorescence) or non-radiative (internal conversion) processes. There's limited time for other processes, such as intersystem crossing to the triplet state or photochemical reactions, to occur.
* **Long-Lived Excited States:** If an excited state has a long lifetime (microseconds to seconds), the molecule has more time to undergo other processes, such as phosphorescence, energy transfer to other molecules, or participate in photochemical reactions.
2. Applications in Fluorescence-Based Techniques:
* **Fluorescence Lifetime Imaging Microscopy (FLIM):** This technique measures the lifetime of excited states to provide information about the local environment of fluorophores, such as pH, viscosity, and proximity to quenchers.

https://www.leica-microsystems.com/science-lab/what-is-flim-fluorescence-lifetime-imaging/
* **Förster Resonance Energy Transfer (FRET):** The efficiency of FRET depends on the lifetime of the donor fluorophore's excited state. By measuring changes in the donor's lifetime, FRET can be used to study interactions between molecules.
https://portlandpress.com/emergtoplifesci/article/5/1/49/228336/Fluorescence-resonance-energy-transfer-in
3. Photochemical Reactions:
* **Reaction Kinetics:** The lifetime of excited states is directly linked to the rates of photochemical reactions. Longer-lived excited states have a higher probability of undergoing reactions before relaxing back to the ground state.
* **Product Formation:** Understanding the lifetime of excited states can help predict the types and yields of photoproducts formed in a reaction.
4. Material Properties:
* **Photostability:** The lifetime of excited states can influence the photostability of materials. Materials with short-lived excited states are generally more photostable, as they dissipate absorbed energy quickly through fluorescence or non-radiative decay.
* **Light-Emitting Devices:** The lifetime of excited states in materials used for light-emitting diodes (LEDs) and other devices is crucial for their efficiency and performance.
In summary, the lifetime of an excited state is not just an abstract concept but a fundamental property that influences various photophysical and photochemical processes. It provides valuable insights into molecular behavior, enables various fluorescence-based techniques, and plays a crucial role in designing and optimizing light-emitting materials.

Meta AI
The lifetime of an excited state is important for the following reasons [1] [2]:
1. Energy-time uncertainty principle: The lifetime of a state is inversely proportional to the uncertainty in the energy.
2. Quantum state: The lifetime of a system in an excited state is usually short, and spontaneous or induced emission of a quantum of energy usually occurs shortly after the system is promoted to the excited state, returning the system to a state with lower energy.
3. Excitation: Excitation refers to an increase in energy level above a chosen starting point, usually the ground state, but sometimes an already excited state.
4. Reaction: A further consequence of excited-state formation may be reaction of the atom or molecule in its excited state, as in photochemistry.
5. Excited-state absorption: The excitation of a system from one excited state to a higher-energy excited state with the absorption of a photon is called excited-state absorption.
6. Calculations: Excited states are often calculated using coupled cluster, Moller-Plesset perturbation theory, multi-configurational self-consistent field, configuration interaction, and time-dependent density functional theory.
The lifetime of an excited state is important as it determines the duration of the excited state and the rate of transition to a lower energy state.
Please let me know if you have any other questions or need further assistance!
https://www.physicsforums.com/threads/lifetime-of-an-excited-state.476199/
https://en.wikipedia.org/wiki/Excited_state

Q7: Why does the fluorescence quantum yield of a fluorophore decrease as the fluorescence spectrum shifts to longer wavelength?

ChatGPT 3.5

The decrease in fluorescence quantum yield as the fluorescence spectrum shifts to longer wavelengths is primarily due to the phenomenon known as the Stokes shift. The Stokes shift refers to the energy difference between the absorption and emission maxima of a fluorophore, and it is a fundamental property of fluorescence spectroscopy.

Here's why the Stokes shift contributes to the decrease in fluorescence quantum yield as the emission wavelength shifts to longer wavelengths:

1. **Increased Vibrational Relaxation**: As the emission wavelength shifts to longer wavelengths, the energy of the emitted photons decreases. This decrease in photon energy corresponds to a decrease in the energy gap between the excited state and the ground state of the fluorophore. Consequently, there is a higher probability of vibrational relaxation processes occurring within the excited state, where excess energy is dissipated as heat through molecular vibrations rather than being emitted as fluorescence photons. This non-radiative decay pathway competes with fluorescence emission, leading to a decrease in fluorescence quantum yield.

2. **Reduction in Radiative Decay Rate**: The radiative decay rate of the excited state, which determines the rate of fluorescence emission, is inversely proportional to the lifetime of the excited state. As the emission wavelength shifts to longer wavelengths, the radiative decay rate typically decreases due to factors such as decreased transition dipole moment, reduced oscillator strength, and increased overlap between the excited state and lower-energy vibrational states. Consequently, the fluorescence lifetime of the excited state becomes longer, increasing the likelihood of non-radiative decay pathways and decreasing the fluorescence quantum yield.

3. **Enhanced Intersystem Crossing**: In some cases, as the emission wavelength shifts to longer wavelengths, there may be an increased propensity for intersystem crossing (ISC) to occur. ISC is a process by which the excited singlet state undergoes transition to the lower-lying triplet state, which is generally longer-lived and less likely to undergo fluorescence emission. The occurrence of ISC competes with radiative decay processes, leading to a reduction in fluorescence quantum yield.

Overall, the decrease in fluorescence quantum yield as the fluorescence spectrum shifts to longer wavelengths is attributed to a combination of factors, including increased vibrational relaxation, reduction in radiative decay rate, and enhanced intersystem crossing, which collectively contribute to the loss of emitted fluorescence photons and the redistribution of energy within the excited state.

ChatGPT 4o

The fluorescence quantum yield of a fluorophore can decrease as the fluorescence spectrum shifts to longer wavelengths due to several factors related to the molecular and environmental properties of the fluorophore. Here are the key reasons:

1. **Energy Gap Law**: The energy gap law states that the rate of non-radiative decay processes increases as the energy gap between the excited state and the ground state decreases. As the fluorescence emission shifts to longer wavelengths (lower energy), the energy gap between the excited state and the ground state becomes smaller. This smaller energy gap facilitates non-

radiative decay processes, such as internal conversion and vibrational relaxation, which compete with radiative decay (fluorescence), thereby reducing the quantum yield.
2. **Increased Non-Radiative Relaxation**: Longer wavelength emissions typically involve lower energy excited states. These lower energy states often have more accessible vibrational modes that can couple with the electronic transitions, leading to increased non-radiative relaxation pathways. The presence of more vibrational modes enhances the probability of energy dissipation through vibrational relaxation, reducing the quantum yield of fluorescence.
3. **Solvent and Environmental Effects**: The local environment and solvent interactions can influence the fluorescence properties of a fluorophore. Solvent molecules can interact more strongly with lower energy excited states, leading to enhanced non-radiative relaxation. Solvent polarity, viscosity, and temperature can all affect the extent of these interactions, contributing to a decrease in quantum yield as the emission wavelength shifts to longer wavelengths.
4. **Intersystem Crossing**: Longer wavelength emissions may also be associated with increased rates of intersystem crossing (ISC), where the fluorophore transitions from an excited singlet state to a triplet state. Triplet states typically have longer lifetimes and are more prone to non-radiative decay processes, such as phosphorescence or non-radiative relaxation. The increased ISC rate can lead to a decrease in fluorescence quantum yield.
5. **Structural Relaxation**: Some fluorophores may undergo significant structural relaxation in the excited state, leading to changes in molecular conformation. These structural changes can result in lower energy emissions and increased non-radiative decay pathways. The structural relaxation processes often become more pronounced at lower energy states, contributing to a decrease in fluorescence quantum yield.
6. **Reabsorption and Re-emission**: In concentrated solutions or environments where reabsorption of emitted light is significant, longer wavelength emissions may be reabsorbed and re-emitted by nearby fluorophore molecules. This reabsorption and re-emission process can introduce additional non-radiative losses, reducing the overall fluorescence quantum yield.
Overall, the decrease in fluorescence quantum yield as the emission spectrum shifts to longer wavelengths is primarily due to the increased likelihood of non-radiative decay processes, enhanced solvent and environmental interactions, increased intersystem crossing, and structural relaxation effects. These factors collectively reduce the efficiency of radiative decay (fluorescence) and contribute to the observed decrease in quantum yield.

Copilot
The fluorescence quantum yield of a fluorophore decreases as the fluorescence spectrum shifts to longer wavelengths due to several factors:
1. Stokes Shift: When a fluorophore absorbs a photon of light, an energetically excited state is formed [1]. The energy loss is due to vibrational relaxation while in the excited state [3]. This shift toward longer wavelengths is called a Stokes shift [3].
2. Energy Loss: Due to the loss of energy by internal conversion of the molecules in the excited state, emitted fluorescence photons are less energetic than the absorbed photons [4]. Accordingly, they have longer wavelengths (lower frequencies), and the fluorescence light color is red-shifted [4].
3. Planck's Law: Planck's Law dictates that the radiation energy of an absorbed photon is directly proportional to the frequency and inversely proportional to the wavelength [5]. This means that shorter incident wavelengths possess a greater quantum of energy [5].

These factors contribute to the decrease in the fluorescence quantum yield as the fluorescence spectrum shifts to longer wavelengths. It's important to note that the exact decrease can vary depending on the specific fluorophore and the conditions of the experiment [1] [2].
Source: Conversation with Copilot, 5/26/2024
A Guide to Recording Fluorescence Quantum Yields
Fluorescence - Chemistry LibreTexts
Fluorescence: General Aspects | SpringerLink
Basic Concepts in Fluorescence - Olympus Microscopy
3.6: Variables that Influence Fluorescence Measurements

Gemini Advanced
The decrease in fluorescence quantum yield (Φf) of a fluorophore as its emission spectrum shifts to longer wavelengths can be attributed to several factors:
1. **Energy Gap Law:** The Energy Gap Law states that the rate of non-radiative decay (k_nr) from the excited state to the ground state increases exponentially as the energy gap between these states decreases.
https://www.nature.com/articles/s41467-023-40716-w
As the fluorescence spectrum shifts to longer wavelengths, the energy gap between the excited and ground states decreases, leading to an increased rate of non-radiative decay. This results in a decrease in the fluorescence quantum yield, as more excited molecules decay through non-radiative pathways rather than emitting a photon.
2. **Increased Vibrational Overlap:** As the emission wavelength increases, the energy difference between the excited and ground states becomes smaller. This leads to a greater overlap between the vibrational levels of the two states, facilitating non-radiative decay processes such as internal conversion and vibrational relaxation. These processes compete with fluorescence, leading to a lower quantum yield.
3. **Solvent Effects:** Solvents can interact with fluorophores and influence their excited state properties.
https://www.olympus-lifescience.com/en/microscope-resource/primer/java/jablonski/solventeffects/
In some cases, solvents with stronger interactions (e.g., polar solvents) can stabilize the excited state and reduce the energy gap, leading to increased non-radiative decay and a lower quantum yield.
4. **Structural Factors:** The molecular structure of the fluorophore can also play a role. Flexible molecules may undergo conformational changes in the excited state, leading to increased non-radiative decay pathways and lower quantum yields.
Overall, the decrease in fluorescence quantum yield with increasing emission wavelength is a complex phenomenon that depends on multiple factors. Understanding these factors is essential for designing and optimizing fluorescent probes and materials with desired properties.
I hope this explanation is helpful! Let me know if you have any other questions.

Meta AI

The fluorescence quantum yield of a fluorophore decreases as the fluorescence spectrum shifts to longer wavelengths due to several factors:

1. Internal conversion: As the fluorescence spectrum shifts to longer wavelengths, the energy gap between the excited state and the ground state decreases, leading to increased internal conversion (non-radiative relaxation). This reduces the fluorescence quantum yield.
2. Vibrational relaxation: Longer wavelengths correspond to smaller energy gaps, allowing for more efficient vibrational relaxation, which competes with fluorescence emission, reducing the quantum yield.
3. Solvent effects: Longer wavelengths often result in increased solvent interaction, leading to enhanced non-radiative relaxation pathways, decreasing the fluorescence quantum yield.
4. Radiative rate reduction: As the fluorescence spectrum shifts to longer wavelengths, the radiative rate (k_r) decreases due to the inverse relationship between wavelength and transition probability, leading to a reduced fluorescence quantum yield.
5. Increased intersystem crossing: Longer wavelengths may facilitate increased intersystem crossing (ISC) to the triplet state, reducing the fluorescence quantum yield.

These factors contribute to the general trend of decreasing fluorescence quantum yield as the fluorescence spectrum shifts to longer wavelengths. However, it’s important to note that specific fluorophores may exhibit varying degrees of these effects, and some may even exhibit increased quantum yields at longer wavelengths due to unique molecular properties.

Q8: Why is the quantum yield of phosphorescence generally low for organic compounds in solution?

ChatGPT 3.5

The quantum yield of phosphorescence is generally low for organic compounds in solution due to several factors:

1. **Spin Selection Rules**: Phosphorescence involves a transition from an excited triplet state to the ground singlet state, which is a spin-forbidden process according to the selection rules of quantum mechanics. This spin-forbiddenness significantly reduces the probability of radiative decay from the triplet state to the ground state, resulting in a low probability of phosphorescence emission.
2. **Non-Radiative Decay Pathways**: In addition to radiative decay (phosphorescence), the excited triplet state can undergo non-radiative decay pathways such as intersystem crossing (ISC) and internal conversion (IC). These pathways involve transitions to lower energy electronic states of the same spin multiplicity (e.g., singlet or triplet states) without emission of a photon. Non-radiative decay processes are often more favorable than radiative decay, particularly in solution environments where interactions with solvent molecules and vibrational modes are prevalent. As a result, a significant fraction of excited triplet states undergo non-radiative decay, reducing the overall quantum yield of phosphorescence.
3. **Quenching Effects**: Various factors in solution, such as molecular collisions, solvent molecules, impurities, and molecular oxygen, can act as quenchers of phosphorescence. These quenching processes can deactivate the excited triplet state through mechanisms such as energy transfer, electron transfer, or collisional relaxation. Molecular oxygen, for example, is a potent quencher of phosphorescence through a process known as triplet oxygen quenching.
4. **Intramolecular Vibrational Relaxation**: In solution, molecules undergo rapid vibrational relaxation due to interactions with the surrounding solvent molecules. Vibrational relaxation dissipates excess energy from the excited state, decreasing the probability of radiative decay and contributing to the low quantum yield of phosphorescence.
5. **Solvent Effects**: The choice of solvent can influence the quantum yield of phosphorescence. Solvents with high viscosity or high polarity can enhance non-radiative decay pathways and increase the rate of quenching, leading to lower phosphorescence quantum yields.

Overall, the low quantum yield of phosphorescence for organic compounds in solution is a consequence of spin selection rules, efficient non-radiative decay pathways, quenching effects, intramolecular vibrational relaxation, and solvent interactions. These factors collectively limit the efficiency of phosphorescence emission in solution environments.

ChatGPT 4o

The quantum yield of phosphorescence for organic compounds in solution is generally low due to several factors:

1. **Spin-Forbidden Transition**: Phosphorescence involves the transition from the triplet excited state to the singlet ground state, which is a spin-forbidden process. This spin-forbidden nature makes the radiative transition (emission of a photon) from the triplet state to the ground

state inherently less probable compared to spin-allowed transitions (such as fluorescence, which involves singlet-singlet transitions).
2. **Non-Radiative Decay Processes**: In solution, organic molecules can undergo various non-radiative decay processes that compete with phosphorescence. These processes include internal conversion (IC) and intersystem crossing (ISC) back to the ground state, as well as vibrational and rotational relaxation. These non-radiative pathways efficiently dissipate the energy of the triplet state without the emission of photons, thereby reducing the quantum yield of phosphorescence.
3. **Quenching by Solvent Molecules**: In solution, solvent molecules can interact with the excited triplet state of the organic compound, leading to quenching. Quenching processes such as collisional quenching, solvent-induced spin-orbit coupling, and energy transfer to solvent molecules can significantly decrease the lifetime of the triplet state and reduce the probability of phosphorescence.
4. **Oxygen Quenching**: Molecular oxygen, which is commonly present in solution, is highly effective at quenching triplet states due to its paramagnetic nature and triplet ground state. Oxygen can efficiently interact with the triplet excited state of the organic compound, facilitating non-radiative deactivation through energy transfer or collisional quenching. This oxygen quenching is one of the primary reasons for the low quantum yield of phosphorescence in solution.
5. **Thermal Deactivation**: In solution, the thermal energy of the environment can promote non-radiative deactivation of the triplet state. The collisions and interactions between solvent molecules and the organic compound can provide sufficient energy to facilitate non-radiative transitions, reducing the likelihood of phosphorescence.
6. **Structural Rigidity**: Phosphorescence is more likely to be observed in rigid matrices or at low temperatures, where the molecular motions are restricted, and non-radiative pathways are minimized. In solution, the flexibility and mobility of organic molecules allow for more efficient non-radiative relaxation, leading to a lower phosphorescence quantum yield.
To observe significant phosphorescence from organic compounds, it is often necessary to use techniques that minimize these quenching effects, such as deoxygenation of the solution, using heavy atom-containing solvents or matrices to enhance spin-orbit coupling, or performing measurements at low temperatures to reduce thermal deactivation and molecular motion.

Copilot
The quantum yield of phosphorescence is generally low for organic compounds in solution due to several factors:
1. Forbidden Transitions: According to quantum mechanics, the transition from the triplet excited state to the singlet ground state is "forbidden" [4]. This means that the rate of this transition is several orders of magnitude smaller than that for fluorescence [4], which results in a lower quantum yield for phosphorescence [4].
2. Aggregation-Caused Quenching (ACQ): In many photoluminescent systems, chromophore aggregation generally quenches light emission [3]. This means that it is necessary to use and study fluorophores in dilute solutions or as isolated molecules [3]. This in turn results in poor sensitivity of devices employing fluorescence, e.g., biosensors and bioassays [3].
3. Self-Quenching: In some cases, such as in crystals of pure chromophore, self-quenching can occur, which reduces the quantum yield [6].
In summary, the low quantum yield of phosphorescence in organic compounds in solution can be attributed to the forbidden nature of the transition from the triplet state to the ground state, aggregation-caused quenching, and self-quenching.

Source: Conversation with Copilot, 5/26/2024
Basics of Fluorescence and Photophysics | SpringerLink
4.5: Photoluminescence, Phosphorescence, and ... - Chemistry LibreTexts
https://www.nature.com/articles/nchem.984.pdf?WT.ec_id
Fluorescence and Phosphorescence - Chemistry LibreTexts
15.1: Theory of Fluorescence and Phosphorescence
Activating efficient phosphorescence from purely organic ... - Nature

Gemini Advanced
The quantum yield of phosphorescence for organic compounds in solution is generally low due to several competing processes that can depopulate the triplet excited state (T1) before phosphorescence can occur. These processes include:
1. **Non-radiative Decay:**
 - **Internal Conversion:** The triplet state can undergo internal conversion, a non-radiative transition to a lower energy singlet state, typically the ground state (S0). This process is often facilitated by vibrational overlap between the two states.
 - **Intersystem Crossing Back to S1:** The triplet state can also undergo intersystem crossing back to the excited singlet state (S1), from which fluorescence or non-radiative decay can occur.
https://www.physicsforums.com/threads/energy-of-singlet-state-and-triplet-state.980860/
2. **Quenching:**
 - **Molecular Oxygen:** Dissolved oxygen is a highly efficient quencher of triplet states.
https://www.researchgate.net/publication/239203826_Mechanism_of_the_excited_singlet_and_triplet_states_quenching_by_molecular_oxygen_in_acetonitrile
It can undergo triplet-triplet annihilation with the excited molecule, transferring energy and returning both molecules to their ground states.
 - **Solvent Interactions:** Interactions with solvent molecules can also quench the triplet state through energy transfer or collisional deactivation.
3. **Photochemical Reactions:**
 - **Triplet Reactivity:** The triplet state is often more reactive than the singlet state due to its longer lifetime and biradical character. This can lead to photochemical reactions that compete with phosphorescence.
4. **Spin-Orbit Coupling:**
 - **Weak Spin-Orbit Coupling:** In most organic molecules, spin-orbit coupling (the interaction between the electron's spin and its orbital motion) is weak. This makes the spin-forbidden transition from the triplet state to the singlet ground state less probable, resulting in a low phosphorescence quantum yield.
To enhance phosphorescence, several strategies can be employed:
* **Low Temperatures:** Lowering the temperature reduces molecular collisions and slows down non-radiative decay processes, increasing the probability of phosphorescence.
* **Rigid Matrices:** Embedding the molecule in a rigid matrix, such as a frozen solvent or polymer, restricts molecular motions and reduces collisions, leading to enhanced phosphorescence.
* **Heavy Atom Effect:** Introducing heavy atoms (e.g., bromine or iodine) into the molecule or the solvent increases spin-orbit coupling, making the triplet-singlet transition more allowed and increasing the phosphorescence quantum yield.
https://en.wikipedia.org/wiki/Phosphorescence

* **Removal of Oxygen:** Degassing the solution to remove dissolved oxygen minimizes quenching and enhances phosphorescence.
By understanding and controlling these factors, researchers can optimize conditions for observing and studying phosphorescence in organic molecules.

Meta AI
Here are the reasons why the quantum yield of phosphorescence is generally low for organic compounds in solution [1]:
1. Singlet and Triplet Excited State: The transition from singlet to triplet (or reverse) involves a change in electronic state, which makes it improbable and leads to a longer lifetime for the triplet state.
2. Jablonski Diagrams: Absorption transitions can occur from the ground singlet electronic state (So) to various vibrational levels in the singlet excited vibrational states. It is unlikely that a transition from the ground singlet electronic state to the triplet electronic state because the electron spin is parallel to the spin in its ground state.
3. Absorption and Emission Rates: The rate of photon absorption is very rapid. Fluorescence emission occurs at a slower rate. Since the triplet to singlet (or reverse) is a forbidden transition, meaning it is less likely to occur than the singlet-to-singlet transition, the rate of triplet to singlet is typically slower.
4. Deactivation Processes: A molecule that is excited can return to the ground state by several combinations of mechanical steps. The deactivation process of fluorescence and phosphorescence involve an emission of a photon radiation.
5. Variables that affect Fluorescence: Molecular structure and its chemical environment influence whether a substance will fluoresce and the intensities of these emissions. The quantum yield or quantum efficiency is used to measure the probability that a molecule will fluoresce or phosphoresce.
6. Phosphorescent Quantum Efficiency: Phosphorescent quantum efficiency is the opposite of fluorescence in that it occurs in the $n \rightarrow \pi^*$ excited state which tends to be short-lived and less susceptible to deactivation than the $\pi \rightarrow \pi^*$ triplet state. Intersystem crossing is also more probable for $\pi \rightarrow \pi^*$ excited state than for the $n \rightarrow \pi^*$ state because the energy difference between the singlet and triplet state is large and spin-orbit coupling is less likely to occur.
In summary, the quantum yield of phosphorescence is generally low for organic compounds in solution due to the improbability of transitions between singlet and triplet states, absorption and emission rates, deactivation processes, and variables affecting fluorescence and phosphorescence quantum efficiency.
https://chem.libretexts.org/Bookshelves/Physical_and_Theoretical_Chemistry_Textbook_Maps/Supplemental_Modules_(Physical_and_Theoretical_Chemistry)/Spectroscopy/Electronic_Spectroscopy/Fluorescence_and_Phosphorescence

Responses to Query 9 (Practical matter)

Q9: What is the inner filter effect and how to avoid it?

ChatGPT 3.5

The inner filter effect (IFE) occurs when a sample absorbs light at the excitation wavelength or emits light at the emission wavelength, leading to a decrease in the measured fluorescence signal. This effect can distort fluorescence measurements, especially in samples with high absorbance at the excitation or emission wavelengths.

Here's how it works and some strategies to avoid it:

1. **Mechanism**: When the excitation light passes through the sample, it can be absorbed by molecules in the sample, reducing the amount of light available for excitation. Similarly, emitted fluorescence light can be absorbed by the sample, reducing the detected fluorescence signal. This absorption by the sample itself is the inner filter effect.

2. **Avoiding the Effect**:

 - **Dilution**: One of the simplest ways to minimize the inner filter effect is to dilute the sample. By reducing the concentration of absorbing molecules in the sample, the extent of light absorption is reduced, mitigating the inner filter effect.

 - **Matching Pathlengths**: Ensure that the pathlengths for absorption and fluorescence measurements are matched. If the pathlengths are significantly different, the inner filter effect can be more pronounced. Using cuvettes with matching pathlengths for both absorption and fluorescence measurements helps minimize this discrepancy.

 - **Correction**: In some cases, it may be possible to correct for the inner filter effect mathematically. This can involve measuring the absorbance of the sample at the excitation and emission wavelengths and applying correction factors to the fluorescence data.

 - **Use of Filters**: Employing optical filters that selectively transmit excitation and emission wavelengths while blocking other wavelengths can help minimize the inner filter effect. These filters help prevent excitation light from reaching the detector during fluorescence measurements and reduce the detection of scattered excitation light.

 - **Choose Appropriate Wavelengths**: Select excitation and emission wavelengths that minimize absorption by the sample. This may involve choosing wavelengths where the sample has lower absorbance or where the inner filter effect is less pronounced.

By employing these strategies, researchers can minimize the inner filter effect and obtain more accurate fluorescence measurements from samples with high absorbance.

ChatGPT 4o

The inner filter effect (IFE) is a phenomenon that can affect fluorescence measurements, particularly in concentrated solutions or samples with high absorbance. It occurs when the excitation or emission light is absorbed by the sample itself, leading to a decrease in the observed fluorescence intensity. There are two types of inner filter effects: primary and secondary.

1. **Primary Inner Filter Effect**: This occurs when the excitation light is absorbed by the sample before it can excite the fluorescent molecules. As a result, fewer photons are available to excite the fluorophores, leading to a reduction in the observed fluorescence intensity.

2. **Secondary Inner Filter Effect**: This occurs when the emitted fluorescence is reabsorbed by the sample before it reaches the detector. This can lead to a distortion of the fluorescence spectrum and a decrease in the observed fluorescence intensity.
How to Avoid the Inner Filter Effect
To minimize or avoid the inner filter effect, several strategies can be employed:
1. **Dilution**: Diluting the sample reduces the concentration of absorbing species, thereby minimizing the probability of reabsorption of excitation or emission light. This is one of the simplest and most effective methods.
2. **Front-Face Illumination**: Instead of measuring fluorescence through the sample (in a right-angle configuration), use front-face illumination where the excitation and emission are measured from the same side. This reduces the path length through which the light travels, minimizing the inner filter effect.
3. **Using Short Path Length Cuvettes**: Reducing the path length of the cuvette (e.g., using a cuvette with a shorter width) decreases the distance the light travels through the sample, thereby reducing the absorption and reabsorption effects.
4. **Corrective Calculations**: Apply mathematical corrections to account for the inner filter effect. These corrections typically involve using the absorbance data at the excitation and emission wavelengths to correct the fluorescence intensity. The correction can be performed using formulas such as:
\[
F_{\text{corrected}} = F_{\text{observed}} \times 10^{(A_{\text{ex}} + A_{\text{em}})/2}
\]
where \(F_{\text{corrected}} \) is the corrected fluorescence intensity, \(F_{\text{observed}} \) is the observed fluorescence intensity, \(A_{\text{ex}} \) is the absorbance at the excitation wavelength, and \(A_{\text{em}} \) is the absorbance at the emission wavelength.
5. **Using Appropriate Solvents and Buffers**: Choose solvents and buffers that do not absorb significantly at the excitation and emission wavelengths to minimize additional absorbance that could contribute to the inner filter effect.
By carefully considering these strategies, one can minimize the inner filter effect and obtain more accurate and reliable fluorescence measurements.

Copilot
The Inner Filter Effect is a phenomenon in fluorescence spectroscopy that results in a loss of observed fluorescence intensity due to the absorption of light by the sample [1]. It is closely linked to the path that the light takes through the sample before it reaches the detector [1]. There are two types of inner filter effects:
1, Primary Inner Filter Effect: As the excitation light travels through the sample, some of it is absorbed, decreasing the intensity the further into the sample it gets. This means that with more absorbance, the detection volume is exposed to less light, leading to a lower degree of excitation and thus less fluorescence [1]. The primary inner filter effect is unavoidable, as a certain amount of light needs to be absorbed for fluorescence to occur [1].
2. Secondary Inner Filter Effect: The emitted light can also get absorbed on its way to the detector, resulting in the secondary inner filter effect [1]. The presence of a secondary inner filter effect will vary greatly depending on the sample composition [1].

The inner filter effect can result in spectral distortion and in some cases complete loss of signal [3]. It becomes a problem when the loss of intensity due to the inner filter effect varies between measurements [1].
To avoid the inner filter effect, the most common approach is to work with concentrations low enough for the effect to be negligible [1]. As a rule of thumb, an optical density of < 0.1 at the excitation wavelength produces good results [1] [2] [3]. If that is not sufficient, the following can be attempted [2]:
*Choose a different excitation wavelength so that the sample absorbance is reduced. It is recommended to move the excitation to 10-50 nm below the absorption maximum to avoid secondary inner filter effects [2].
*Use a low-volume cuvette or a triangular cuvette. This reduces the path length and in turn the sample absorbance [2].
*In some spectrometers, the cuvette can be placed in a front-face sample holder. This holder is designed for solid samples but accommodates cuvettes. It allows to detect the emission at the same surface illuminated by the excitation beam, which reduces the path length even further [2].
Source: Conversation with Copilot, 5/26/2024
What is the Inner Filter Effect and How Does it Impact Fluorescence ...
What Is The Inner Filter Effect? | Environmental XPRT
What is the Inner Filter Effect? | Edinburgh Instruments | Blog

Gemini Advanced
The inner filter effect (IFE) occurs in fluorescence spectroscopy and can significantly impact the accuracy of fluorescence measurements. It's caused by the absorption of both excitation and emission light by the fluorophore itself or other components in the sample.
https://pubs.acs.org/doi/10.1021/acs.analchem.2c01031
There are two types of inner filter effects:
1. **Primary Inner Filter Effect:** This occurs when the excitation light is absorbed by the fluorophore or other absorbing species before it reaches the region of the sample being observed.
https://www.labbot.bio/blog-posts/what-is-the-inner-filter-effect-and-how-does-it-impact-fluorescence-measurements
This reduces the excitation intensity, leading to a decrease in the observed fluorescence signal.
2. **Secondary Inner Filter Effect:** This occurs when the emitted fluorescence is reabsorbed by the fluorophore or other absorbing species before it can escape the sample and reach the detector.
https://www.edinst.com/blog/inner-filter-effect/
This also results in a decrease in the observed fluorescence signal and can also distort the shape of the emission spectrum.
How to Avoid or Minimize the Inner Filter Effect:
1. **Reduce sample concentration:** The most common approach is to dilute the sample to a point where the absorbance at both the excitation and emission wavelengths is low (ideally below 0.1).
https://www.edinst.com/blog/inner-filter-effect/
This reduces the probability of absorption occurring before the light reaches the desired region or the detector.
2. **Choose a different excitation wavelength:** If diluting the sample is not possible, try using a different excitation wavelength where the sample absorbance is lower.
3. **Use a smaller pathlength cuvette:** This reduces the distance the light has to travel through the sample, decreasing the chances of absorption.

4. **Mathematical corrections:** In some cases, mathematical corrections can be applied to compensate for the inner filter effect.
https://pubs.acs.org/doi/10.1021/acs.analchem.2c01031
These corrections are based on the sample's absorbance at the excitation and emission wavelengths.
5. **Use specialized cuvettes or microplate readers:** Special cuvettes, like triangular or low-volume cuvettes, can minimize the path length of excitation and emission light, reducing IFE. Some microplate readers can also adjust the focus to minimize IFE.
Important Considerations:
* It's crucial to be aware of the inner filter effect when working with fluorescence measurements, as it can lead to significant errors in data interpretation.
https://www.labbot.bio/blog-posts/what-is-the-inner-filter-effect-and-how-does-it-impact-fluorescence-measurements
* Always check the absorbance of your sample at both the excitation and emission wavelengths to assess the potential impact of IFE.
I hope this explanation is helpful! Let me know if you have any other questions.

Meta AI
The inner filter effect (IFE) is a phenomenon in spectroscopy where the absorbance of a sample affects the fluorescence or phosphorescence emission intensity. It occurs when the emitted light is absorbed by the sample itself or by impurities in the sample, leading to a reduction in the detected emission intensity.
Causes of inner filter effect:
1. Self-absorption: The sample absorbs its own emitted light, reducing the detected intensity.
2. Impurity absorption: Impurities in the sample absorb the emitted light, contributing to the IFE.
Consequences of inner filter effect:
1. Reduced emission intensity: Lower detected emission intensity, potentially leading to incorrect conclusions about the sample's properties.
Distorted spectra: Altered spectral shapes and peak ratios, making it challenging to interpret the data accurately.
Ways to avoid or minimize the inner filter effect:
1. Dilute the sample: Reduce the concentration of the sample to minimize self-absorption and impurity absorption.
2. Use a smaller sample volume: Decrease the path length of the emitted light through the sample, reducing absorption.
3. Choose a different solvent: Select a solvent with minimal absorbance at the emission wavelength.
4. Use an inner filter correction: Apply mathematical corrections to account for the IFE, if possible.
5. Optimize instrument settings: Adjust the instrument's slit widths, detector sensitivity, and excitation intensity to minimize the IFE.
6. Use a front-face fluorescence geometry: Align the excitation and emission paths to minimize the light path through the sample.
7. Purify the sample: Remove impurities that contribute to the IFE.
By understanding and addressing the inner filter effect, you can obtain more accurate and reliable spectroscopic data.

Responses to Query 10 (Philosophical issue)

Q10: Please create the most important photochemistry questions.

ChatGPT 3.5

Certainly! Here are some important questions in the field of photochemistry:

1. What are the fundamental principles underlying the absorption of light by molecules, and how does it lead to electronic excitation and the formation of excited states?
2. How do the selection rules of quantum mechanics govern the transitions between electronic states, and what are the implications of these rules for photochemical processes?
3. What are the mechanisms of radiative and non-radiative decay pathways in excited states, and how do they influence the overall quantum yield of fluorescence, phosphorescence, and other photophysical processes?
4. How do molecular structure, electronic configuration, and environmental factors (e.g., solvent polarity, temperature, pH) influence the photophysical properties and reactivity of photoactive molecules?
5. What are the different types of photochemical reactions, such as photoisomerization, photodissociation, and energy transfer processes, and what factors control their efficiency and selectivity?
6. How can photochemical reactions be harnessed for practical applications, including solar energy conversion, photovoltaics, photocatalysis, photodynamic therapy, and molecular imaging?
7. What are the mechanisms of photodegradation and photochemical aging in materials, polymers, and organic compounds, and how can these processes be mitigated or controlled?
8. How can advanced spectroscopic techniques, such as time-resolved spectroscopy, fluorescence lifetime imaging microscopy (FLIM), and transient absorption spectroscopy, be used to study ultrafast photophysical and photochemical processes on femtosecond to millisecond timescales?
9. What are the recent advances in the design and synthesis of photoresponsive materials, including photochromic compounds, fluorescent probes, and optoelectronic devices, and how do they enable new functionalities and applications?
10. How can computational methods, quantum chemical calculations, and theoretical models be used to predict and rationalize the photophysical properties, excited state dynamics, and reaction mechanisms of photoactive molecules?

These questions cover a broad range of topics in photochemistry, from the fundamental principles of light-matter interactions to the practical applications of photochemical processes in various fields of science and technology.

ChatGPT 4o

Certainly! Here are some important questions in the field of photochemistry:

1. **Fundamental Concepts**:
   - What is photochemistry, and how does it differ from thermal chemistry?
   - What are the basic principles of the Jablonski diagram, and how do they describe the electronic states and transitions of molecules?

2. **Electronic Transitions**:
- What are the differences between singlet and triplet states, and how do their properties influence photochemical processes?
- How do absorption and emission spectra relate to the electronic transitions in molecules?
3. **Fluorescence and Phosphorescence**:
- What are the mechanisms of fluorescence and phosphorescence, and how do they differ?
- What factors influence the quantum yield of fluorescence and phosphorescence?
4. **Photophysical Processes**:
- What are the primary non-radiative decay processes (e.g., internal conversion, intersystem crossing), and how do they affect the fate of excited states?
- How do the lifetimes of excited states impact photochemical reactions and applications?
5. **Photochemical Reactions**:
- What are common types of photochemical reactions, such as photoisomerization, photodissociation, and photoreduction?
- How does the energy of absorbed light drive chemical reactions in photochemistry?
6. **Excitation and Emission**:
- What is the significance of the Stokes shift in fluorescence spectroscopy?
- How do inner filter effects and quenching influence the measurement of fluorescence?
7. **Photostability and Degradation**:
- What mechanisms lead to photodegradation of molecules, and how can these processes be mitigated?
- How does the stability of a photochemical system affect its practical applications?
8. **Applications of Photochemistry**:
- How is photochemistry applied in fields such as photovoltaics, photocatalysis, and photodynamic therapy?
- What role do photosensitizers play in photochemical reactions, particularly in medical and environmental applications?
9. **Environmental Photochemistry**:
- How do photochemical processes in the atmosphere influence environmental phenomena, such as the formation of smog and the degradation of pollutants?
- What is the role of sunlight in driving photochemical reactions in natural waters?
10. **Advanced Techniques**:
- What are the key techniques used to study photochemical processes, such as time-resolved spectroscopy and fluorescence lifetime imaging microscopy (FLIM)?
- How do computational methods contribute to the understanding of photochemical mechanisms and the design of photochemical systems?
11. **Energy Transfer**:
- What are the mechanisms of energy transfer in photochemical systems, such as Förster resonance energy transfer (FRET) and Dexter energy transfer?
- How do energy transfer processes influence the efficiency and outcome of photochemical reactions?
12. **Molecular Design**:
- How does the structure of a molecule influence its photochemical behavior, including absorption characteristics, excited state dynamics, and reactivity?
- What strategies are used in the design of photochemically active materials, such as organic dyes, semiconductors, and photosensitizers?

These questions cover a broad range of topics within photochemistry, from fundamental principles to practical applications and advanced research techniques.

Copilot

Sure, here are some important questions related to photochemistry:

1. What is photochemistry? Can you explain the basic principles of photochemistry?
2. What is the difference between the ground state and the excited state of a molecule?
3. What is the Jablonski diagram? How does it represent the various electronic states and transitions in a molecule?
4. What is the difference between fluorescence and phosphorescence? How does the lifetime of the excited state affect these processes?
5. What is the Franck-Condon principle? How does it explain the mirror image relationship between absorption and emission spectra?
6. What is the Stokes shift? Why is it important in fluorescence spectroscopy?
7. What is the inner filter effect in fluorescence spectroscopy? How can it be minimized or corrected?
8. What is the role of the triplet state in photochemistry? Why is it generally longer-lived than the singlet state?
9. What is quantum yield? How is it calculated and what factors can affect it?
10. What are some applications of photochemistry in real-world scenarios? Can you provide examples in areas such as solar energy, medicine, and environmental science?

These questions cover a broad range of topics in photochemistry and can help deepen understanding of this field. Each question opens up discussion on key concepts and principles that are fundamental to photochemistry.

Gemini Advanced

Absolutely! Here are some of the most important questions in photochemistry, covering fundamental concepts, experimental techniques, and applications:

Fundamental Concepts:

1. What are the primary laws of photochemistry (Grotthuss-Draper law, Stark-Einstein law)?
2. What is the Jablonski diagram and how does it illustrate photophysical processes?
3. What are the different types of electronic transitions in molecules (e.g., singlet-singlet, singlet-triplet)?
4. How does the Franck-Condon principle influence absorption and emission spectra?
5. What is the difference between fluorescence and phosphorescence?
6. How does the solvent environment affect the photophysical properties of molecules?
7. What are the main mechanisms of photochemical reactions (e.g., electron transfer, energy transfer, bond cleavage)?
8. How does the quantum yield determine the efficiency of a photochemical process?
9. What is the importance of the excited state lifetime in photochemistry?
10. What are the key factors influencing the rate of a photochemical reaction?

Experimental Techniques:

1. What are the common methods for measuring absorption and emission spectra?
2. How can fluorescence lifetimes and quantum yields be determined experimentally?
3. What are the principles and applications of transient absorption spectroscopy?
4. How can flash photolysis be used to study short-lived intermediates in photochemical reactions?

5. What are the advantages and limitations of different light sources (e.g., lasers, lamps) for photochemical studies?
Applications:
1. How is photochemistry used in solar energy conversion (e.g., photovoltaics, artificial photosynthesis)?
2. What are the photochemical processes involved in vision and other biological systems?
3. How does photochemistry contribute to environmental issues (e.g., photodegradation of pollutants, ozone depletion)?
4. What are the applications of photochemistry in materials science (e.g., photoresists, photopolymerization)?
5. How is photochemistry used in organic synthesis (e.g., photocycloadditions, photoisomerizations)?
6. What are the recent advances and future directions in photoredox catalysis?
7. How can photochemistry be used for controlled drug delivery and release?
8. What are the challenges and opportunities in developing photoresponsive materials for various applications?
I hope these questions provide a comprehensive overview of the key areas in photochemistry!

Meta AI
Here are some of the most important photochemistry questions:
1. What is the difference between fluorescence and phosphorescence?
2. How does the energy of light influence chemical reactions?
3. What is the role of the excited state in photochemical reactions?
4.How do molecules absorb light, and what are the factors that affect absorption?
5. What is the significance of the Stokes shift in photochemistry?
6. How do photochemical reactions differ from thermal reactions?
7. What are the applications of photochemistry in fields like medicine, materials science, and environmental science?
8. How do photosensitizers and photocatalysts work, and what are their uses?
9. What is the importance of quantum yield in photochemical reactions?
10. How do solvent effects and molecular interactions influence photochemical reactions?
11. What are the differences between singlet and triplet excited states, and how do they affect photochemical reactions?
12. How does spin conservation influence photochemical reactions?
13. What are the mechanisms of photochemical reactions, such as electron transfer and energy transfer?
14. How do photochemical reactions affect the structure and properties of molecules?
15. What are the current research areas and advancements in photochemistry?
These questions cover fundamental concepts, mechanisms, and applications of photochemistry, and answering them can provide a comprehensive understanding of the field.

Responses to Queries 11–13 (Dyes and fluorophores)

Q11: Why are azo dyes non-fluorescent?

ChatGPT 3.5

Azo dyes are typically non-fluorescent due to several factors inherent in their molecular structure and electronic properties:

1. **Electron Delocalization**: Azo dyes contain the azo (-N=N-) functional group, which consists of two nitrogen atoms connected by a double bond. The presence of this conjugated system allows for extensive electron delocalization along the molecular backbone. As a result, the electronic transitions responsible for fluorescence are often quenched or destabilized due to the presence of nearby energy levels.
2. **Internal Conversion and Intersystem Crossing**: Azo dyes may undergo rapid internal conversion and intersystem crossing processes, leading to efficient non-radiative decay pathways. Internal conversion involves the conversion of electronic energy into vibrational energy within the same electronic state, while intersystem crossing involves the transition between different electronic states of different spin multiplicities. These processes compete with fluorescence, leading to the loss of electronic energy without emission of photons.
3. **Energy Dissipation**: In many cases, the excited states of azo dyes have relatively short lifetimes due to efficient energy dissipation mechanisms. Vibrational relaxation, rotational relaxation, and collisional quenching processes can rapidly dissipate excess energy from the excited state, preventing the accumulation of sufficient energy for fluorescence emission.
4. **Electronic Configuration**: The electronic configuration of azo dyes may not be conducive to fluorescence. Depending on the specific molecular structure and substituent groups, the electronic transitions responsible for fluorescence may be energetically unfavorable or prohibited by selection rules, leading to the absence of fluorescence emission.
5. **Solvent Effects**: The fluorescence properties of azo dyes can be influenced by the surrounding solvent environment. Polar solvents or solvents with high viscosity may enhance non-radiative decay pathways and decrease the efficiency of fluorescence emission. Conversely, non-polar solvents may stabilize the excited state and promote fluorescence emission, but this effect is typically minimal for azo dyes.

Overall, the non-fluorescent nature of azo dyes is a consequence of their molecular structure, electronic properties, and the presence of efficient non-radiative decay pathways. While some azo dyes may exhibit weak fluorescence under specific conditions, the majority of azo dyes are non-fluorescent under ambient conditions.

ChatGPT 4o

Azo dyes are generally non-fluorescent due to several key reasons related to their molecular structure and photophysical properties:

1. **Efficient Non-Radiative Decay Pathways**: Azo dyes have highly conjugated structures that facilitate efficient non-radiative decay pathways. These pathways, such as internal conversion and vibrational relaxation, allow the excited state energy to be dissipated as heat rather than being

emitted as fluorescence. The presence of azo groups (–N=N–) in these dyes promotes non-radiative relaxation processes, which significantly quench fluorescence.
2. **Intersystem Crossing to Triplet State**: The nitrogen-nitrogen double bond in azo dyes can promote intersystem crossing (ISC), a process where the excited singlet state transitions to an excited triplet state. The triplet state typically has a much longer lifetime and can undergo non-radiative decay more easily than radiative decay (phosphorescence). The efficient ISC reduces the probability of fluorescence emission from the singlet excited state.
3. **Planar and Rigid Structure**: The planar and rigid structure of azo dyes leads to strong electronic interactions and extensive π-π stacking. This structural characteristic can facilitate non-radiative decay mechanisms and hinder the molecule's ability to emit fluorescence. In addition, the rigid structure can lead to close packing in solid states or aggregates in solution, further enhancing non-radiative decay pathways.
4. **Photochemical Stability**: Azo dyes are prone to photochemical reactions, such as photoisomerization, where the azo group undergoes a trans-cis isomerization upon absorption of light. This isomerization can provide a pathway for non-radiative relaxation and further reduces the likelihood of fluorescence emission. The isomerization process typically results in a significant change in the dye's electronic structure, leading to quenching of fluorescence.
5. **Heavy Atom Effect**: In some azo dyes, the presence of heavy atoms (such as iodine or bromine) can enhance spin-orbit coupling, which facilitates intersystem crossing and increases non-radiative decay. The heavy atom effect promotes the transition from the singlet excited state to the triplet state, reducing the fluorescence quantum yield.
Due to these factors, azo dyes typically exhibit low fluorescence quantum yields, making them non-fluorescent or weakly fluorescent. Their primary application lies in their strong absorption properties, which make them useful as colorants in various industries, rather than their emission properties.

Copilot
Azo dyes are typically non-fluorescent due to their molecular structure and the nature of their electronic transitions. Here are some key reasons:
1. Azo Group Structure: Azo dyes contain the azo group (-N=N-), which allows for the absorption of light [1]. However, the absorption peak of azobenzene, a common azo dye, is in the UV region [3]. This means it only absorbs a small amount of visible light, which is not enough to exhibit fluorescence.
2. Non-radiative Decay Pathways: After absorbing light, molecules can return to the ground state through various pathways. In the case of azo dyes, the excited state molecule can react with oxygen [4]. This non-radiative decay pathway competes with fluorescence, reducing the likelihood of a fluorescent emission.
3. Fluorescent Probes: Due to the non-fluorescent nature of azo dyes, fluorescent probes are often used to track their pathways. These probes are attached to the azo dye by an alkyl bond [2].
It's important to note that while azo dyes are generally non-fluorescent, there may be exceptions depending on the specific dye structure and the conditions of the experiment. Always consult relevant scientific literature or a knowledgeable colleague if you're unsure.
Source: Conversation with Copilot, 5/26/2024
Azo dye - Wikipedia
aromatic compounds - What is the origin of the colour of azo dyes ...
A new way to improve the light-fastness of azo reactive dyes through ...

Degradation of Synthetic Azo Dyes of Textile Industry: a ... - Springer
http://www.ibef.org/industry/textiles.aspx
en.wikipedia.org

Gemini Advanced
Azo dyes are generally non-fluorescent due to the presence of the azo (-N=N-) group in their structure. This group facilitates efficient non-radiative decay pathways that compete with fluorescence:
1. **Photoisomerization:** The azo group readily undergoes *cis-trans* photoisomerization upon excitation.
https://pubs.acs.org/doi/10.1021/acs.joc.2c00661
This process involves a twisting motion around the N=N bond, dissipating the absorbed energy as heat rather than emitting it as fluorescence.
2. **Internal Conversion:** The azo group also promotes internal conversion, a non-radiative transition from the excited state to the ground state through vibrational relaxation. This process further reduces the likelihood of fluorescence.
3. **n-π* Transitions:** Azo dyes often have low-lying n-π* excited states, which are characterized by weak absorption and efficient non-radiative decay. The presence of these states further contributes to the lack of fluorescence.
4. **Charge Transfer:** In some azo dyes, the excited state can undergo intramolecular charge transfer, where electron density shifts from one part of the molecule to another. This process can also lead to non-radiative decay and quenching of fluorescence.
However, there are exceptions to this general rule. Some azo dyes exhibit weak fluorescence under specific conditions, such as low temperatures or rigid environments that restrict photoisomerization. Additionally, researchers have developed some fluorescent azo compounds by modifying the molecular structure to suppress non-radiative decay pathways.
I hope this explanation is helpful! Let me know if you have any other questions.

Meta AI
Azo dyes are non-fluorescent due to the following reasons [1 2]:
1. Fluorescence quenching: Azo-bone of azo dyes has fluorescen quenching, which results in deep depression of fluorescence intensity for azo dyes.
2. Weak fluorescence intensity: Fluorescence intensity of typic azo dyes is very weak.
3. Breakdown of azo-bone: When azo-bone of azo dyes is broken down by electro-fenton, their fluorescence intensity increases dramatically, and this phenomenon is the unique features of azo dyes.
4. Low fluorescence efficiency: Fluorescent azobenzene derivatives have quite low fluorescence efficiencies.
Please note that there are some azo dyes that can be fluorescent, such as Congo Red [3].
https://en.wikipedia.org/wiki/Azo_dye
https://www.researchgate.net/publication/275872736_Fluorescence_Characteristics_of_Typic_Azo_and_Non-Azo_Dyes
https://expert.cheekyscientist.com/7-non-fluorescent-histology-dyes/

Q12: Why do porphyrins typically exhibit two peaks in the fluorescence spectrum?

ChatGPT 3.5

Porphyrins typically exhibit two peaks in the fluorescence spectrum due to the presence of multiple electronic states and vibrational sublevels within the porphyrin molecule. This phenomenon is commonly observed and can be attributed to several factors:

1. **Electronic Transitions**: Porphyrins contain a highly conjugated system of alternating single and double bonds, which gives rise to a complex electronic structure with multiple electronic transitions. The fluorescence spectrum of porphyrins often shows two distinct peaks corresponding to different electronic transitions, such as the Soret band and the Q bands.
2. **Soret Band**: The Soret band is a high-energy absorption band observed at shorter wavelengths in the UV-visible spectrum of porphyrins. Fluorescence emission from the Soret band typically occurs at shorter wavelengths and is characterized by a higher energy peak in the fluorescence spectrum.
3. **Q Bands**: The Q bands are a series of lower-energy absorption bands observed at longer wavelengths in the UV-visible spectrum of porphyrins. Fluorescence emission from the Q bands occurs at longer wavelengths and is characterized by a lower energy peak in the fluorescence spectrum.
4. **Vibrational Sublevels**: Each electronic state in the porphyrin molecule is associated with multiple vibrational sublevels due to molecular vibrations. The interaction between electronic and vibrational states leads to vibrational progression in the fluorescence spectrum, resulting in additional peaks or shoulder peaks in the fluorescence spectrum.
5. **Solvent Effects and Aggregation**: The presence of solvent molecules or aggregation effects can also influence the fluorescence spectrum of porphyrins. Changes in solvent polarity, viscosity, or aggregation state can affect the relative intensities and positions of the fluorescence peaks, leading to variations in the fluorescence spectrum.

Overall, the presence of multiple electronic transitions, vibrational sublevels, and environmental factors contribute to the complex fluorescence spectrum observed in porphyrins, typically characterized by two distinct peaks corresponding to the Soret band and the Q bands. Understanding the origins of these fluorescence peaks is crucial for interpreting porphyrin fluorescence spectra and studying their photophysical properties.

ChatGPT 4o

Porphyrins typically exhibit two peaks in the fluorescence spectrum due to their characteristic electronic structure and the nature of their excited states. The two peaks are often referred to as the Q-bands, and their occurrence can be explained as follows:

1. **Electronic Transitions**: Porphyrins have a highly conjugated macrocyclic structure that leads to a complex electronic configuration with several closely spaced energy levels. The primary electronic transitions responsible for absorption and fluorescence in porphyrins occur between the ground state (S0) and the first excited singlet states (S1) and involve two main sets of transitions called the Q-bands and the B-band (or Soret band). The Q-bands arise from transitions between the S0 ground state and the S1 excited state, which are split into two closely spaced energy levels due to the symmetry of the porphyrin ring.

2. **Vibrational Structure**: The two fluorescence peaks in the Q-band region correspond to transitions from the lowest vibrational levels of the first excited singlet state (S1) to different vibrational levels of the ground state (S0). The higher energy Q-band peak (Q(0,0)) typically corresponds to the transition between the zero vibrational level of the excited state and the zero vibrational level of the ground state. The lower energy Q-band peak (Q(1,0)) corresponds to the transition from the zero vibrational level of the excited state to a higher vibrational level of the ground state.
3. **Franck-Condon Principle**: The relative intensities and positions of the Q-band peaks are influenced by the Franck-Condon principle, which describes the probability of electronic transitions between different vibrational states. The Franck-Condon factors depend on the overlap of vibrational wavefunctions between the excited and ground states. This overlap determines the relative intensities of the Q(0,0) and Q(1,0) transitions, leading to the observed two-peak structure in the fluorescence spectrum.
4. **Solvent Effects and Environmental Factors**: The exact positions and intensities of the Q-band peaks can be influenced by the solvent environment, solvent polarity, and interactions with surrounding molecules. These factors can cause slight shifts and variations in the fluorescence spectrum but generally do not change the fundamental two-peak structure.
In summary, the two peaks in the fluorescence spectrum of porphyrins are due to the electronic transitions between the ground and excited states, with the presence of vibrational sublevels in these states leading to multiple fluorescence peaks. This characteristic spectral feature is a result of the unique electronic structure and symmetry of the porphyrin ring.

Copilot

Porphyrins typically exhibit two peaks in the fluorescence spectrum due to the nature of their electronic transitions. Here's why:
1. Soret Band (B Band): This is a strong transition to the second excited state (S0 → S2) at about 400 nm [2]. This band is intense and is often seen in the blue region of the spectrum [2].
2. Q Band: This is a weak transition to the first excited state (S0 → S1) at about 550 nm [2]. This band is less intense and is often seen in the red region of the spectrum [2].
These transitions arise from the π-π* transitions in the porphyrin molecule [2]. The Gouterman four-orbital model explains these transitions by considering the two highest occupied molecular orbitals (HOMOs) and the two lowest unoccupied molecular orbitals (LUMOs) of the porphyrin molecule [2]. Mixing of these states splits them in energy, creating a higher energy 1 eu state with greater oscillator strength (Soret band) and a lower energy 1 eu state with less oscillator strength (Q-bands) [2].
Internal conversion from S2 to S1 is rapid, so fluorescence is only detected from S1 [2]. This results in the characteristic two-peak fluorescence spectrum of porphyrins. Please note that the exact positions and intensities of these bands can vary depending on the specific porphyrin and its environment [2].
Source: Conversation with Copilot, 5/26/2024
Absorption and Fluorescence Spectroscopy of and Metallo ...
Fluorescence of Porphyrins in Cells | SpringerLink
3.6: Variables that Influence Fluorescence Measurements
The Light Spectrum and its Relationship with Fluorescence

Gemini Advanced

Porphyrins typically exhibit two peaks in their fluorescence spectrum due to the unique electronic structure and vibrational transitions associated with their macrocyclic ring system.

Electronic Structure:

* **Q bands:** The two peaks in the fluorescence spectrum of porphyrins are often referred to as the Q bands (Qx and Qy). These bands arise from transitions between the ground electronic state (S0) and the first excited singlet state (S1) of the porphyrin molecule.

* **Degenerate States:** The S1 state of porphyrins is actually a pair of closely spaced, nearly degenerate energy levels (Qx and Qy).

https://pubs.acs.org/doi/10.1021/acs.jpclett.0c00856

This degeneracy arises from the high symmetry of the porphyrin ring.

* **Vibrational Transitions:** Each of the Qx and Qy electronic transitions is further split into multiple vibrational bands due to the various vibrational modes of the molecule. The most prominent vibrational transitions are often the 0-0 (purely electronic) and 0-1 transitions, which correspond to the two main peaks observed in the fluorescence spectrum.

https://www.nature.com/articles/srep22756

Q Band Intensity:

The relative intensities of the Qx and Qy bands can vary depending on the specific porphyrin derivative and its environment. Factors such as the substituents on the porphyrin ring, the solvent, and the presence of metal ions can influence the electronic structure and thus the relative intensities of the Q bands.

Overall:

The two peaks in the fluorescence spectrum of porphyrins are a consequence of the electronic structure of the porphyrin ring, which results in two closely spaced, nearly degenerate excited states (Qx and Qy). Each of these states further undergoes vibrational transitions, giving rise to the two distinct peaks observed in the spectrum.

Meta AI

Porphyrins exhibit two peaks in the fluorescence spectrum due to the following reasons [1]:

1. pH-dependent variation in fluorescence intensity: Porphyrins exhibit a pH-dependent variation in fluorescence intensity, which leads to the appearance of multiple peaks in the fluorescence spectrum.
2. Three-peak profile: At certain pHs, a clear three-peak profile is observed, which is designated as peaks a, β, and γ.
3. Effect of ionic strength: The fluorescence intensity of porphyrins also depends on ionic strength, reaching sharp maxima at 0.1 mol/L (for uroporphyrin) and 1 mol/L (for coproporphyrin).
4. Solvent composition: The organic mixture ethyl acetate:acetic acid (4:1 by vol), commonly used to extract porphyrins from biological samples, markedly diminishes the fluorescence of both porphyrins as compared with the same concentration of each porphyrin in aqueous acidic solvent.

In summary, porphyrins exhibit two peaks in the fluorescence spectrum due to pH-dependent variation in fluorescence intensity, ionic strength, and solvent composition. These factors lead to the appearance of multiple peaks in the fluorescence spectrum, which can be used to quantify porphyrins in biological samples.

https://www.researchgate.net/publication/19795933_Factors_influencing_fluorescence_spectra_of_free_porphyrins

Q13: Why do Quantum dots typically exhibit high brightness?

ChatGPT 3.5

Quantum dots (QDs) typically exhibit high brightness due to several unique properties inherent in their nanoscale structure and semiconductor composition:

1. **High Quantum Yield**: Quantum dots have a high quantum yield, which is the efficiency with which they convert absorbed photons into emitted photons. This high quantum yield results from the confinement of excitons (electron-hole pairs) within the nanoscale dimensions of the QD, leading to reduced non-radiative decay pathways and enhanced radiative recombination efficiency.
2. **Size-Tunable Emission**: Quantum dots exhibit size-dependent optical properties, including tunable emission wavelengths. By controlling the size and composition of the quantum dots during synthesis, researchers can precisely tune the energy bandgap and emission wavelength of the QDs. This size-tunable emission enables QDs to emit light at specific wavelengths with narrow spectral linewidths, resulting in high color purity and brightness.
3. **Broad Absorption Spectra**: Quantum dots have broad absorption spectra that span a wide range of wavelengths, from ultraviolet (UV) to near-infrared (NIR) regions of the electromagnetic spectrum. This broad absorption enables efficient excitation of QDs by a wide range of light sources, including UV lamps, lasers, and light-emitting diodes (LEDs), enhancing their brightness and applicability in various optical and imaging applications.
4. **High Photostability**: Quantum dots exhibit high photostability under continuous excitation and prolonged exposure to light. Unlike organic dyes or fluorescent proteins, QDs are less prone to photobleaching and degradation, allowing for long-term and repeated imaging without loss of fluorescence intensity. This high photostability contributes to the sustained brightness of QDs over extended periods of time.
5. **Single-Particle Brightness**: Quantum dots possess high single-particle brightness, meaning that individual QDs emit a high number of photons per unit time. This property arises from the efficient radiative recombination of excitons within the nanoscale volume of each quantum dot. High single-particle brightness is particularly advantageous for single-molecule and single-particle imaging applications, where detection sensitivity is critical.

Overall, the combination of high quantum yield, size-tunable emission, broad absorption spectra, high photostability, and single-particle brightness enables quantum dots to exhibit exceptionally high brightness compared to traditional fluorophores, making them valuable tools for a wide range of biomedical, optoelectronic, and imaging applications.

ChatGPT 4o

Quantum dots (QDs) typically exhibit high brightness due to several key factors related to their unique physical and chemical properties:

1. **High Quantum Yield**: Quantum dots have high quantum yield, which is the ratio of the number of photons emitted to the number of photons absorbed. This high quantum yield results from efficient radiative recombination of electron-hole pairs (excitons) within the quantum dot, leading to intense fluorescence emission.
2. **Size-Tunable Emission**: The emission wavelength of quantum dots can be precisely tuned by changing their size due to the quantum confinement effect. Smaller quantum dots emit light at

shorter wavelengths (blue), while larger quantum dots emit light at longer wavelengths (red). This tunability allows for the selection of quantum dots with optimal emission properties for specific applications, enhancing their brightness.
3. **Narrow Emission Spectra**: Quantum dots exhibit narrow and symmetric emission spectra with a full width at half maximum (FWHM) typically between 20-40 nm. This narrow emission peak results in a high signal-to-noise ratio and bright fluorescence, as most of the emitted light is concentrated within a specific wavelength range.
4. **Photostability**: Quantum dots are more photostable compared to traditional organic dyes. They resist photobleaching and maintain their brightness over extended periods of illumination. This photostability is crucial for applications that require long-term imaging or continuous light exposure, such as in biological imaging and display technologies.
5. **Multiple Excitons Generation**: Quantum dots can sometimes generate multiple excitons (electron-hole pairs) from the absorption of a single high-energy photon. This phenomenon, known as multiple exciton generation (MEG), can enhance the fluorescence intensity, contributing to their high brightness.
6. **Efficient Light Absorption**: Quantum dots have a high molar extinction coefficient, meaning they can absorb light very efficiently. This efficient absorption enhances the probability of exciton formation, leading to increased fluorescence emission and higher brightness.
7. **Surface Passivation**: The surface of quantum dots is often passivated with organic ligands or inorganic shells (such as a ZnS shell around a CdSe core). This passivation reduces surface defects and non-radiative recombination pathways, ensuring that more of the absorbed energy is released as fluorescence, thus increasing the brightness.
8. **Versatility in Functionalization**: Quantum dots can be easily functionalized with various chemical groups, allowing them to be conjugated with biomolecules, polymers, or other materials. This versatility enables their use in a wide range of environments and applications while maintaining high brightness.
Together, these properties make quantum dots exceptionally bright and advantageous for applications in fluorescence imaging, display technologies, solar cells, and other fields requiring strong and stable light emission.

Copilot
Quantum dots typically exhibit high brightness due to their unique physical and chemical properties. Here are some key reasons:
1. Size-Dependent Emission: Quantum dots are semiconductor particles a few nanometers in size, and their size affects the allowed quantum states for electrons and holes [1]. These states determine the energies and therefore the colors of the emitted photons [1]. By producing these nanoparticles in a variety of sizes, researchers can make dots that shine in almost any color [3].
2. High Quantum Yield: Brightness is an important measure for quantum dots and is related to the number of photons the quantum dot emits per second [4]. Quantum dots radiate photons of a specific color (and hence frequency) after being excited, for example, by ultraviolet light of a higher frequency [4].
3. Efficient Light Conversion: In a quantum dot TV, the quantum dots are applied to a film, which replaces a color filter — a filter that usually means the loss of at least some of the light provided by the backlight [2]. With a lot less light lost, the image can be a whole lot brighter [2].

These properties make quantum dots ideal for applications that require high brightness, such as display technologies [2]. However, the exact brightness of a quantum dot can vary depending on its size, shape, and the specific conditions of the experiment [1].
Source: Conversation with Copilot, 5/26/2024
Quantum dot - Wikipedia
Physics - A Quantum Dot Shows Its True Colors
How to make bright quantum dots even brighter - Phys.org
Quantum Dots in QLED TVs: What are they and what do they do?
Getty Images

Gemini Advanced
Quantum dots (QDs) typically exhibit high brightness due to several factors:
1. **High Quantum Yield:** Quantum dots often have very high quantum yields, meaning they convert a large proportion of absorbed light into emitted light.
https://jnanobiotechnology.biomedcentral.com/articles/10.1186/s12951-021-01227-2
This is due to their efficient radiative recombination of electron-hole pairs, resulting in minimal non-radiative energy loss.
2. **Tunable Absorption and Emission:** The size of a quantum dot determines its bandgap energy, which directly influences the wavelengths of light it can absorb and emit.
https://www.news-medical.net/life-sciences/Quantum-Dot-Optical-Properties.aspx
This allows for precise tuning of the absorption and emission properties, maximizing the amount of light that can be absorbed and then re-emitted as fluorescence.
3. **Narrow Emission Spectra:** Quantum dots typically have narrow emission spectra, meaning they emit light over a smaller range of wavelengths compared to traditional fluorophores.
https://www.researchgate.net/figure/Broad-absorption-and-narrow-emission-spectrum-15_fig3_324097340
This concentrated emission results in a higher intensity at the peak wavelength, contributing to increased brightness.
4. **Photostability:** Quantum dots are generally more resistant to photobleaching than traditional fluorophores.
https://pubs.rsc.org/en/content/articlehtml/2015/cs/c4cs00532e
This means they can be excited repeatedly without significant degradation of their fluorescence intensity, allowing for prolonged and brighter emission.
5. **Large Absorption Cross-Section:** Quantum dots have a large absorption cross-section, meaning they can efficiently capture photons from the excitation light source.
This leads to a higher number of excited electron-hole pairs and ultimately a brighter fluorescence emission.
These combined factors contribute to the high brightness of quantum dots, making them attractive materials for various applications, such as display technologies, bioimaging, and solar cells.

Meta AI
Here are the reasons why quantum dots typically exhibit high brightness [1] [2]:
1. Precise Color and Higher Peak Brightness: Quantum dots can be tuned to emit the precise amount of light, which means the color produced will be more accurate. The nanocrystals can render a wider spectrum color at maximum brightness without losing saturation.
2. Better Energy Efficiency: Quantum dots only require a small amount of energy to work, and this can be achieved by a single blue light. In fact, they can produce a 30 percent increase in brightness while using about 30 to 50 percent less power. This reduced power consumption results in lower costs. Unlike conventional LCDs that waste energy combining and balancing different colors, QLED screens emit precise colors for improved energy efficiency.
3. Longer Lifespan and Low Burn-In: As QLED displays are based on existing LCD technology, they benefit from having a longer lifespan as they rely on modern and energy-efficient backlighting. Quantum dot technology also does not degrade over time, meaning QLED displays are less prone to burn-in.
4, Versatile Sizes and Thinner Panels: Since quantum dot technology is easier and less expensive to produce, QLED displays can be made in a greater range of sizes and can go as large as 98 inches. They can also be applied on large, thin, and flexible LCD panels, making the QLEDs easy to transport and install.
Quantum dots are usually applied to a sheet of film that sits as a layer in that “sandwich” in front of the LED backlight that’s used to illuminate an LCD. The light passes through the LCD display stack, with the Quantum Dot color filter layer enhancing and enabling the LCD to reveal a wider and more saturated range of colors than would otherwise be possible.

https://insights.samsung.com/2021/12/29/what-is-quantum-dot-display-technology/
https://www.viewsonic.com/library/tech/explained/what-is-quantum-dot-technology/
